# Evaluating protein prenylation of human and viral CaaX sequences using a humanized yeast system

**DOI:** 10.1242/dmm.050516

**Published:** 2024-05-31

**Authors:** Emily R. Hildebrandt, Anushka Sarkar, Rajani Ravishankar, June H. Kim, Walter K. Schmidt

**Affiliations:** Department of Biochemistry and Molecular Biology, University of Georgia, Athens, GA 30602, USA

**Keywords:** CaaX, Farnesylation, Geranylgeranylation

## Abstract

Prenylated proteins are prevalent in eukaryotic biology (∼1-2% of proteins) and are associated with human disease, including cancer, premature aging and infections. Prenylated proteins with a C-terminal CaaX sequence are targeted by CaaX-type prenyltransferases and proteases. To aid investigations of these enzymes and their targets, we developed *Saccharomyces cerevisiae* strains that express these human enzymes instead of their yeast counterparts. These strains were developed in part to explore human prenyltransferase specificity because of findings that yeast FTase has expanded specificity for sequences deviating from the CaaX consensus (i.e. atypical sequence and length). The humanized yeast strains displayed robust prenyltransferase activity against CaaX sequences derived from human and pathogen proteins containing typical and atypical CaaX sequences. The system also recapitulated prenylation of heterologously expressed human proteins (i.e. HRas and DNAJA2). These results reveal that substrate specificity is conserved for yeast and human farnesyltransferases but is less conserved for type I geranylgeranyltransferases. These yeast systems can be easily adapted for investigating the prenylomes of other organisms and are valuable new tools for helping define the human prenylome, which includes physiologically important proteins for which the CaaX modification status is unknown.

## Teaser

**Summary:** A humanized yeast model system has been developed for investigating CaaX sequence modification by CaaX-type prenyltransferases and proteases, enabling preliminary investigations of previously uncharacterized and physiologically relevant CaaX proteins.

## INTRODUCTION

Post-translational modification of the C-terminal CaaX sequence (herein referring to all C-terminal tetrapeptide sequences regardless of whether they fit the traditional consensus sequence) is important for regulating the localization and function of many proteins, including the Ras GTPases often cited as archetypical CaaX proteins (Campbell and Philips, 2021; Cox et al., 2015; Ravishankar et al., 2023). Ras oncogenic mutants are associated with 90-95% of certain cancers (https://www.cancer.gov/research/key-initiatives/ras), and oncogenic phenotypes are altered when CaaX modification is disrupted (Bergo et al., 2002; Cox et al., 2015; Sousa et al., 2008; Winter-Vann and Casey, 2005). In addition to cancer, CaaX proteins are functionally implicated in numerous other disease states including Alzheimer's disease, progeria, liver disease, and viral infections such as hepatitis D and severe acute respiratory syndrome coronavirus 2 (SARS-CoV-2) (Jeong et al., 2022; Marakasova et al., 2017; Soveg et al., 2021; Wickenhagen et al., 2021; Yang et al., 2012; Zhao et al., 2020). A recent example demonstrating the importance of protein prenylation for viral infection resulted from studies of *OAS1* gene splice variants, which concluded that humans expressing a prenylated variant of the Oas1 protein had higher innate antiviral protection against Sars-CoV-2 than those expressing the non-prenylated protein (Soveg et al., 2021; Wickenhagen et al., 2021). With regard to Alzheimer's disease, the CaaX protein Ydj1 (yeast ortholog of human DNAJA1) has been demonstrated to directly interact with amyloid β42 oligomers and drive amyloid β42 toxicity in model organisms (Ring et al., 2022). Defining the full spectrum of prenylated proteins is thus crucial for understanding the impacts of pharmacological interventions that globally alter prenylation patterns and lead to a combined effect arising from altering the prenylation of many CaaX proteins simultaneously.

**Fig. 1.**
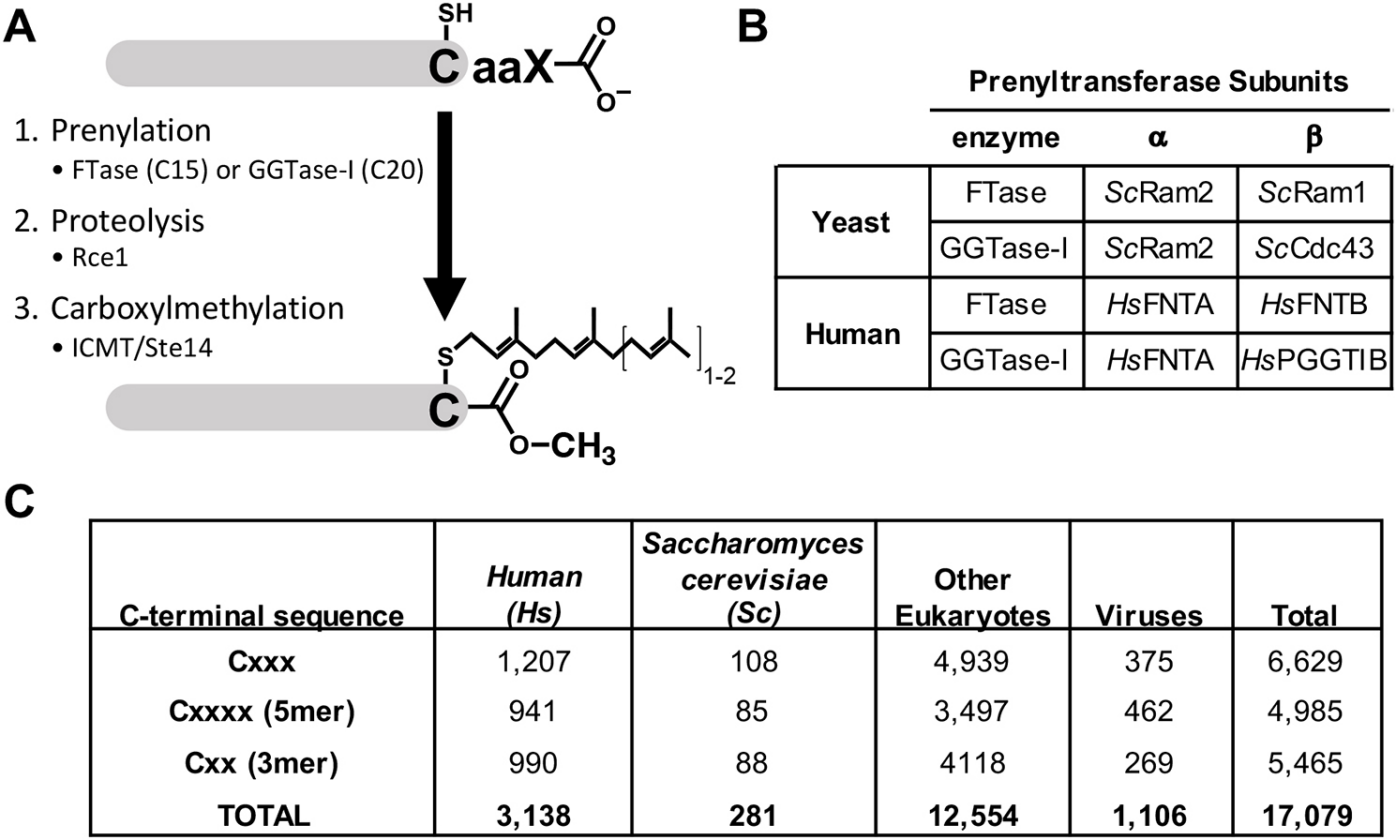
**The CaaX protein modification pathway, prenyltransferase enzymes and CaaX prenylome estimates.** (A) Post-translational modification pathway of CaaX proteins. Many proteins [e.g. human (*Hs*) HRas, KRas, NRas and prelamin A, yeast (*Sc*) **a**-factor, etc.] undergo a three-step modification pathway: (1) cysteine prenylation, (2) proteolysis of the last three amino acids by Rce1 and (3) carboxymethylation at the C-terminus by *Hs*ICMT/*Sc*Ste14. Some proteins (e.g. *Sc*Ydj1 and *Hs*Nap1L1) undergo prenylation without further modification. (B) Relationship between enzyme subunits of yeast (*Sc*) and human (*Hs*) farnesyltransferase (FTase) and geranylgeranyltransferase type I (GGTase-I). (C) Summary of proteins identified in the UniProtKB/Swiss-Prot database with the indicated C-terminal CaaX sequences. Bacteriophage sequences were not included in the number determined for viruses.

The C-terminal CaaX sequence has been traditionally defined as a cysteine (C), two aliphatic amino acids (a_1_a_2_), and one of several amino acids (X) (reviewed in Jung and Bachmann, 2023; Wang and Casey, 2016). The first step in CaaX modification is covalent attachment of a farnesyl (C15) or geranylgeranyl (C20) isoprene lipid to the cysteine. Farnesyltransferase (FTase) generally targets CaaX sequences, whereas geranylgeranyltransferase type I (GGTase-I) targets the subset of CaaL/I/M sequences (Hartman et al., 2005). Some CaaX sequences are modified by both enzymes, albeit one is typically preferred (Caplin et al., 1998; Fiordalisi et al., 2003; Moores et al., 1991; Trueblood et al., 1993). CaaX sequences with an aliphatic amino acid at the a_2_ position commonly follow the canonical pathway that involves two additional modification steps: proteolytic removal of aaX and carboxymethylation of the exposed prenylated cysteine (Fig. 1A). In yeast, the Ras2 GTPase and **a**-factor mating pheromone are well-characterized examples of canonically modified CaaX proteins. Conversely, CaaX sequences with non-aliphatic residues at a_1_ and a_2_ can be farnesylated but resist proteolysis, resulting in a biochemically distinct C-terminus compared to that of canonically modified CaaX proteins (Kim et al., 2023). These so-called shunted CaaX sequences are prevalent and occur throughout biology, with the yeast HSP40 Ydj1 protein being the best-characterized example to date (Hildebrandt et al., 2016b).

FTase and GGTase-I are heterodimeric enzymes. They share an α subunit, whereas the β subunits provide isoprenoid specificity and substrate recognition (Lane and Beese, 2006; Maurer-Stroh et al., 2003). Human FTase [*Homo sapiens* (*Hs*)FTase] is composed of *Hs*FNTA(α) and *Hs*FNTB(β) subunits; the orthologous subunits of yeast FTase [*Saccharomyces cerevisiae* (*Sc*)FTase] are *Sc*Ram2(α) and *Sc*Ram1(β) (Fig. 1B). Human GGTase-I (*Hs*GGTase-I) is composed of *Hs*FNTA(α) and *Hs*PGGT1B(β) subunits, whereas the orthologous subunits of yeast GGTase-I (*Sc*GGTase-I) are *Sc*Ram2(α) and *Sc*Cdc43(β). In yeast, *RAM1* is not an essential gene (i.e. *ram1*Δ strains are viable), whereas *RAM2* and *CDC43* are essential, raising the possibility that GGTase-I is more critical to yeast life processes relative to FTase (He et al., 1991; Ohya et al., 1993). In this study, we describe the manipulation of the yeast genome to allow for expression of human FTase and GGTase-I in lieu of their yeast counterparts.

Using the broadest definition of a CaaX sequence (i.e. cysteine followed by any three amino acids), 1207 human proteins can be identified within UniProtKB/Swiss-Prot. These proteins use 680 of the 8000 possible CaaX sequence combinations. There are also 375 viral proteins with a CaaX sequence that could potentially utilize host enzymes for CaaX modification (Fig. 1C). Resolving the prenylation potential of candidate CaaX sequences has depended on a variety of methods and prediction algorithms, each with its own limitations. Case-by-case methods for verification of protein prenylation include radiolabeling with ^3^H-mevalonate (i.e. the farnesyl and geranylgeranyl precursor) and analyses based on gel mobility, localization and mass spectrometry (Anderegg et al., 1988; Hancock et al., 1989; Hildebrandt et al., 2016b; Michaelson et al., 2005; Ravishankar et al., 2023). Methods for systematic verification have relied on metabolic labeling with reagents compatible with click chemistry, peptide arrays and genetic screens (Kho et al., 2004; Kim et al., 2023; Rashidian et al., 2013; Storck et al., 2019; Suazo et al., 2018, 2021; Wang et al., 2014). Differentiating FTase and GGTase-I target specificity has mostly relied on *in vitro* and genetic approaches (Hougland et al., 2010; Kim et al., 2023; Stein et al., 2015). Independent of the methods used, it remains a distinct challenge to confirm the prenylation status of candidate CaaX sequences, especially in organisms in which prenyltransferase specificities have never been investigated or studied in depth.

In this study, we developed yeast strains expressing *Hs*FTase and *Hs*GGTase-I that functionally replace the yeast enzymes in a wide array of tests. Results with a universal reporter for both farnesylation and geranylgeranylation indicate that human prenyltransferases target a wide variety of human and viral CaaX sequences, with human FTase having an ability to modify non-canonical sequences like its yeast counterpart. We also demonstrate the utility of this model system for evaluating the prenylation of heterologously expressed human proteins. This system provides a valuable new resource for protein prenylation research in a genetically amenable cell-based system. This resource is expected to enhance the identification and characterization of previously unreported prenyltransferase targets, including those harboring atypical CaaX sequences.

## RESULTS

### Interspecies complementation analysis of FTase subunits

**Fig. 2.**
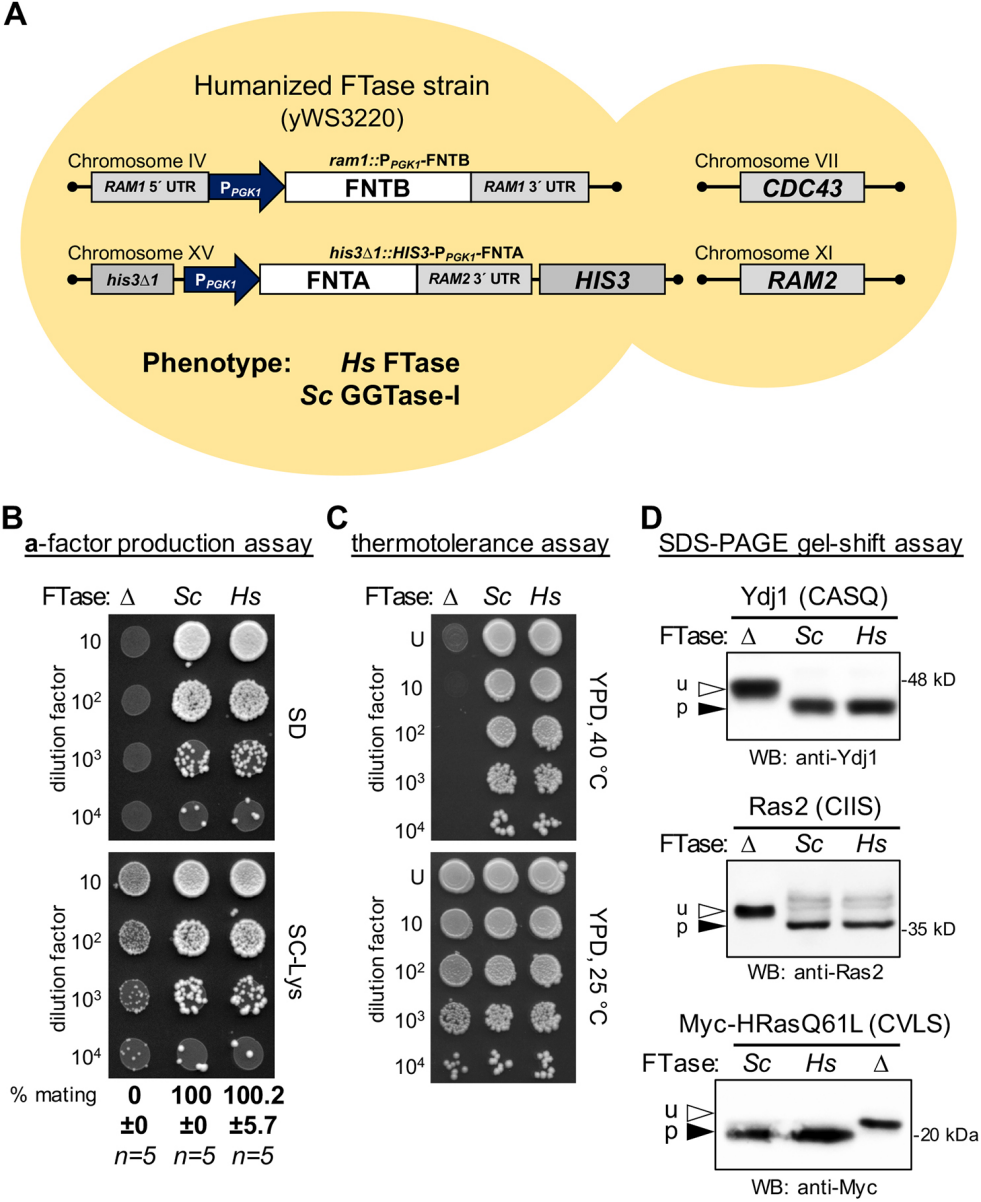
**The humanized *Hs*FTase yeast system is comparable to wildtype yeast in a series of functional tests.** (A) Key genetic features of the humanized yeast *Hs*FTase strain. Coding sequences for *Hs*FTase subunits were integrated into yeast chromosomes. *P_PGK1_*-*FNTA* was inserted at the *HIS3* locus; *P_PGK1_*-*FNTB* replaced the *RAM1* coding sequence. This strain retained *Sc*GGTase-I activity. (B) The mating assay was used to assess production of farnesylated **a**-factor mating pheromone by yeast and human FTase. *MAT***a** haploid cells (i.e. **a**-factor producing) were mixed with *MAT*α haploid cells (SM1068), the mixes subjected to 10-fold dilutions, and dilution mixtures spotted onto minimal (SD) and SC-lysine (SC-Lys) solid media. The genetics of this assay are such that only diploids can grow on SD media. Growth on SC-Lys reflects the input of *MAT***a** cells and serves as a control for the dilution procedure. Quantitative mating test results are reported below each lane relative to wildtype yeast (*Sc*). Errors are standard error of the mean (s.e.m.). The strains used were: yWS3202 (**Δ**; no FTase), BY4741 (wild type, *Sc*) and yWS3220 (*Hs*). (C) The thermotolerance assay was used to compare temperature-dependent FTase activities of indicated strains. The strains are the same as those described in panel B. The experiment was repeated independently multiple times (*n*=5). (D) The gel-shift assay was used to compare FTase activities of the indicated strains. Total cell lysates were prepared from the strains used in panels A and B, and equivalent protein amounts analyzed by western blotting (WB) using anti-Ydj1 (top) or anti-Ras2 (middle) antibody. Lysates were also prepared from strains that were transformed with a plasmid encoding human HRas (p-05547), and the immunoblot was probed with anti-Myc antibody (bottom). The experiments were repeated independently multiple times (Ydj1, *n*=10; Ras2, *n*=3; HRas, *n*=2). u, unprenylated; p, prenylated.

As a key first step toward developing a yeast system to express *Hs*FTase, complementation studies were performed to assess the functional equivalence of yeast and human FTase subunits. Codon-optimized genes encoding *Hs*FNTA(α) and *Hs*FNTB(β) were introduced into yeast, separately or in combination, and evaluated for their ability to complement for loss of the yeast FTase β subunit Ram1 (i.e. *ram1*Δ). For this evaluation, we used a highly sensitive and well-characterized biological assay (i.e. yeast mating) that detects production of the farnesylated **a**-factor mating pheromone (Hildebrandt et al., 2016b; Nijbroek and Michaelis, 1998). There is a direct correlation between **a**-factor levels and mating efficiency, and the high-sensitivity assay can quantifiably measure **a**-factor levels as low as 0.001% relative to normal levels. Co-expression of both *Hs*FNTA(α) and *Hs*FNTB(β) as genome-integrated genes resulted in a mating phenotype that was qualitatively and quantifiably indistinguishable from mating exhibited by wildtype yeast, indicative of normal **a**-factor production (Fig. 2A,B). Similar observations were made with plasmid-encoded genes (Fig. S1A,B, lanes 2 and 5). Importantly, despite observations that human proteins can often substitute for their yeast counterparts, *Hs*FNTB(β) expressed in yeast was unable to support **a**-factor production (i.e. mating), indicative of no prenyltransferase activity (Fig. S1A,B, lane 3). This result indicates either that *Sc*Ram2(α) and *Hs*FNTB(β) subunits do not interact or that a formed hybrid FTase complex [*Sc*Ram2(α)-*Hs*FNTB(β)] is non-functional. During development of these strains, differences in mating efficiency were also observed depending on the promoters used to express the subunits (Fig. S1C). This observation ultimately directed us to integrate human FTase subunits into the yeast genome using the constitutive phosphoglycerate kinase promoter (i.e. *P_PGK1_*) rather than orthologous yeast promoters.

As additional phenotypic confirmation of human FTase activity in yeast, we observed that the temperature-sensitive growth phenotype of the parental FTase-deficient strain (i.e. *ram1*Δ) had been alleviated in the humanized FTase strain (Fig. 2C). To further confirm the ability of yeast-expressed *Hs*FTase to prenylate other CaaX proteins, gel-shift analysis was used to determine the extent of farnesylation for yeast Ydj1, yeast Ras2 and a human oncogenic variant of HRas (Fig. 2D; Fig. S1D). The strains were used directly to prepare cell lysates containing endogenous levels of Ydj1 and Ras2, or they were transformed with a yeast expression plasmid encoding Myc-tagged HRas^Q61L^ to obtain an HRas-containing lysate. A gel shift was observed for all three proteins when *Sc*FTase or *Hs*FTase was present; the faint upper bands associated with farnesylated Ras2 are occasionally observed and are of unknown origin. Each protein exhibited faster gel mobility than that of its unfarnesylated form. The reason for this counterintuitive but common observation remains unknown. Not all prenylated proteins demonstrate a gel shift upon prenylation, and the direction of the shift can vary for different proteins. Nonetheless, these complementation studies indicate that *Hs*FTase engineered into yeast can support the normal prenylation of both yeast and human CaaX proteins.

### *Hs*FTase expressed in yeast modifies human Ras CaaX sequences

**Fig. 3.**
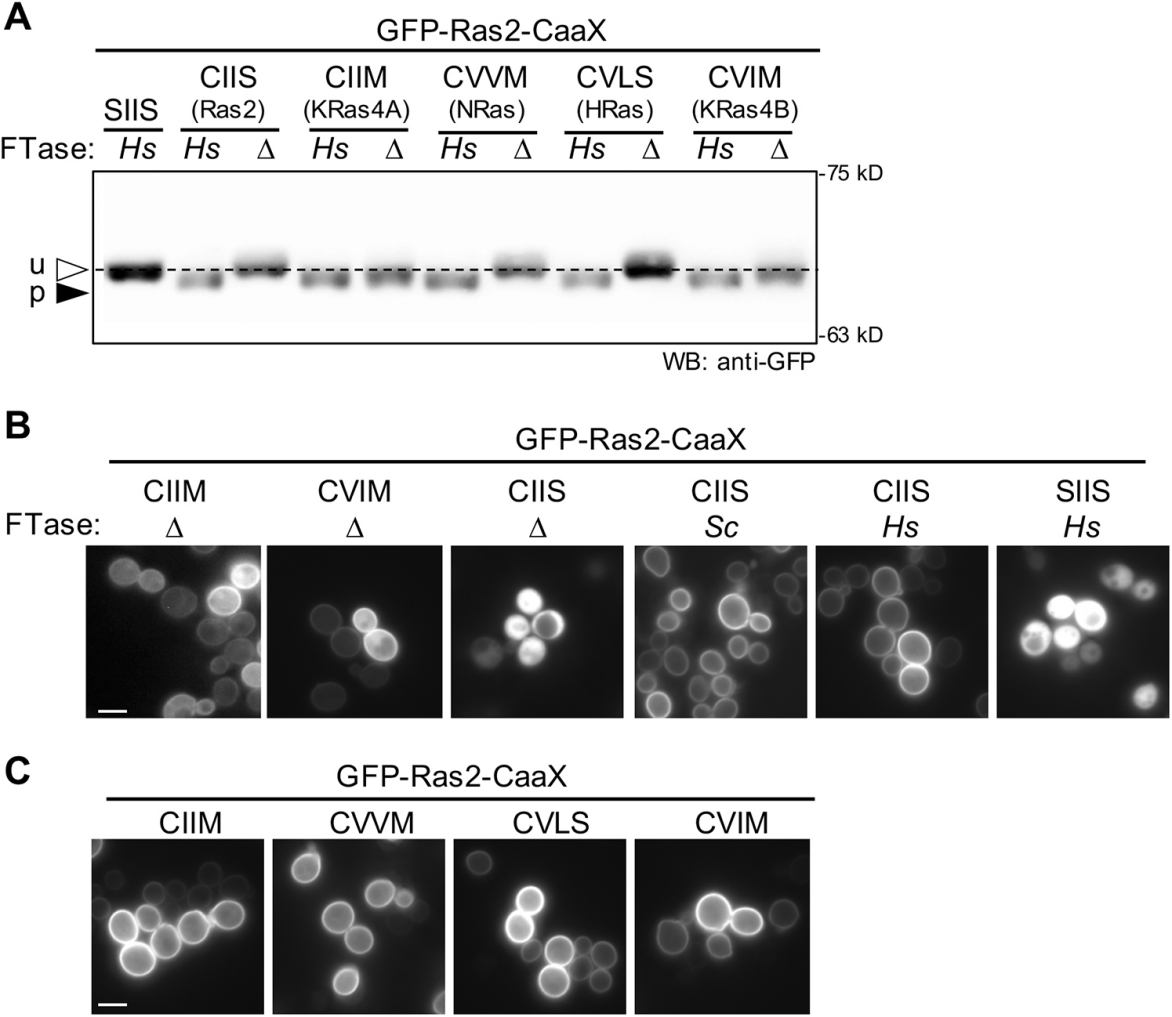
**The humanized *Hs*FTase system farnesylates yeast and human Ras CaaX sequences.** (A) The humanized *Hs*FTase yeast system, GFP-Ras2 reporter and gel-shift assay were used to evaluate farnesylation of CaaX sequences derived from yeast Ras2 and human Ras proteins (KRas4A, NRas, HRas and KRas4B) and an unprenylated control (SIIS). Total cell lysates were prepared and analyzed by immunoblotting as described in Fig. 2D using anti-GFP antibody. The dashed line in the immunoblot image was aligned with the unprenylated GFP-Ras2 band to serve as a visual reference. The experiment was repeated independently multiple times (*n*=3). u, unprenylated; p, prenylated. (B,C) The localization of GFP-Ras2-CaaX variants in yeast was determined by fluorescence microscopy in the absence (Δ) and presence of yeast (*Sc*) and human (*H*s) FTase. CaaX sequences were derived from yeast Ras2 and the human Ras sequences described in panel A. Multiple fields of cells were captured for each strain using at least two biological replicates of each strain. Scale bars: 5 µm. The yeast strains used were: BY4741 (*Sc*), yWS3220 (*Hs*) and yWS3202 (Δ). The plasmids used are listed in Table S4. The GFP-Ras2-SIIS plasmid also contains a mutated cysteine adjacent to CIIS that is typically palmitoylated; it was mutated to avoid having a CaaaX sequence of non-canonical length (i.e. CSIIS).

Our complementation studies revealed that *Hs*FTase can modify a variety of yeast and human CaaX proteins: **a**-factor (with the CaaX sequence CVIA), Ras2 (CIIS), Ydj1 (CASQ) and HRas (CVLS). We next used the humanized *Hs*FTase yeast system, a GFP-Ras2 reporter and gel-shift analysis to investigate farnesylation of human Ras CaaX sequences: KRas4A KRas4A (CIIM) and KRas4B (CVIM) (both encoded by *KRAS*), HRas (CVLS) and NRas (CVVM) (Fig. 3A). In the presence of *Hs*FTase, all the GFP-Ras2-CaaX variants exhibited faster mobility relative to that of a negative control (i.e. GFP-Ras2-SIIS) that cannot be prenylated due to the absence of cysteine within its CaaX sequence. Moreover, the GFP-Ras2-CaaX variants based on HRas (CVLS) and NRas (CVVM) did not shift in the absence of FTase (Δ), whereas those based on KRas4A (CIIM) and KRas4B (CVIM) still shifted. In mammalian systems, some CaaX sequences are prenylated by *Hs*GGTase-I when *Hs*FTase activity is diminished (Mohammed et al., 2016; Whyte et al., 1997). We thus infer that alternate geranylgeranylation occurs for CIIM and CVIM sequences in FTase-deficient yeast that still express endogenous *Sc*GGTase-I.

In yeast and humans, Ras localization to the plasma membrane depends on canonical modification of the CaaX sequence (Boyartchuk et al., 1997; Michaelson et al., 2005; Ravishankar et al., 2023). Bolstering our conclusion that alternate prenylation occurred for GFP-Ras2-CIIM and -CVIM, these proteins exhibited plasma membrane localization in the absence of FTase, which would be predicted if they were geranylgeranylated (Fig. 3B). Importantly, GFP-Ras2-CIIS was only membrane localized in the presence of FTase (either yeast or human), consistent with observations that it is not subject to alternate prenylation, and this localization was dependent on the presence of cysteine within its CaaX sequence as has been previously observed (Dong et al., 2003; Ravishankar et al., 2023). We also used the plasma membrane localization phenotype of the GFP-Ras2 reporter to confirm that *Hs*FTase could modify CaaX sequences associated with the human Ras orthologs (Fig. 3C). Each sequence directed GFP-Ras2 to the plasma membrane, indicating farnesylation by *Hs*FTase. Combined, these observations indicate that mammalian CaaX sequences can be modified by the humanized *Hs*FTase strain and that *Sc*GGTase-I has the opportunistic ability to modify certain CaaX sequences in the absence of FTase activity.

### *Hs*FTase expressed in yeast modifies the non-canonical human DNAJA2 CAHQ sequence

Our complementation studies indicated that *Hs*FTase can modify the Ydj1 CASQ sequence (Fig. 2D). The ability to modify CASQ and a wide-range of non-canonical CaaX sequences, in addition to canonical sequences, is a recently identified property of *Sc*FTase that has not been systematically investigated for FTase from other species (Berger et al., 2018; Hildebrandt et al., 2016b; Kim et al., 2023; Ravishankar et al., 2023).

**Fig. 4.**
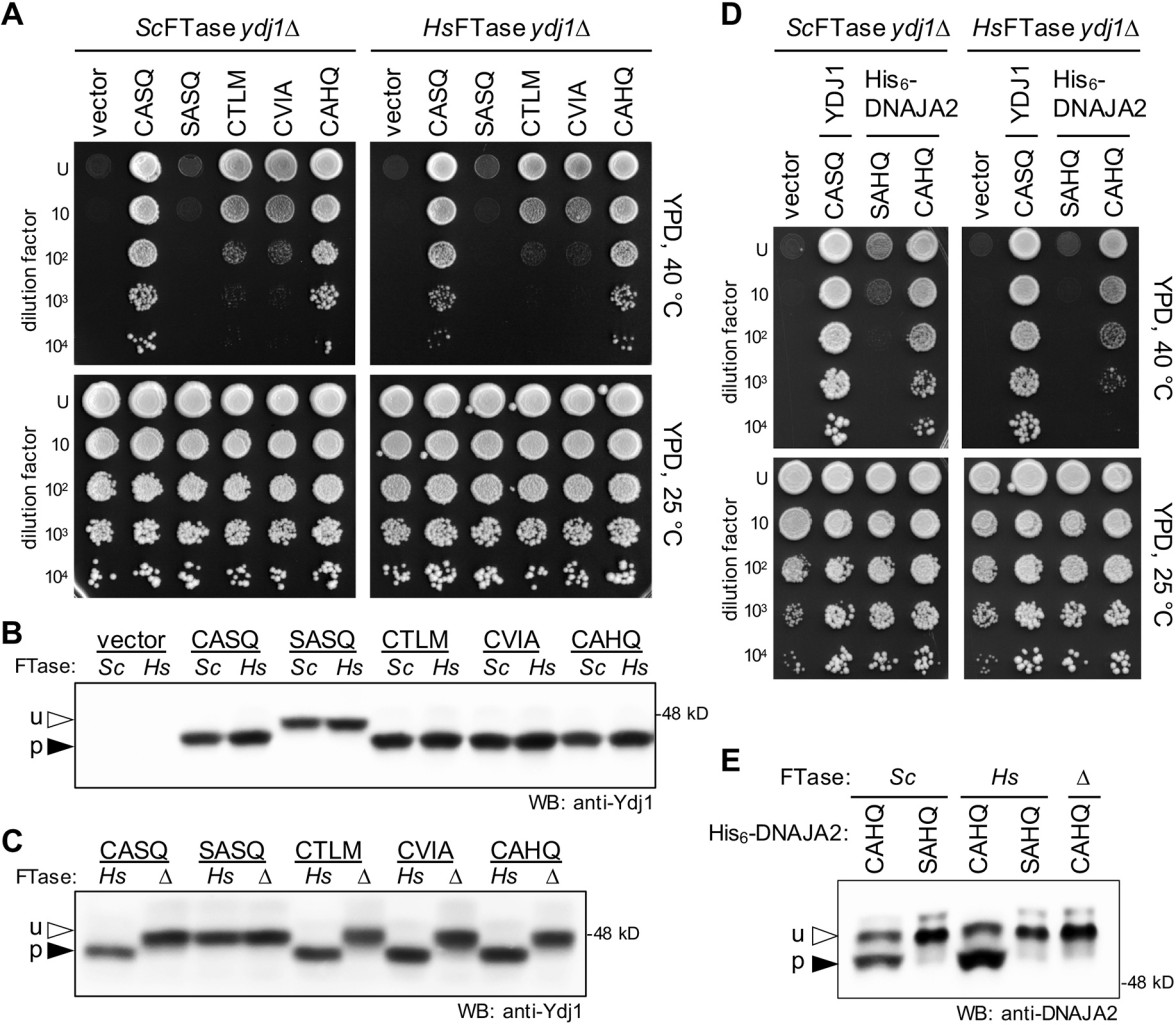
**The humanized *Hs*FTase system modifies Ydj1-CaaX variants.** (A) The humanized *Hs*FTase yeast system, Ydj1-CaaX reporter and thermotolerance assay were used to evaluate farnesylation of CaaX sequences from yeast Ydj1 (CASQ), yeast Gγ Ste18 (CTLM), yeast **a**-factor (CVIA) and human HSP40 DNAJA2 (CAHQ); an unmodifiable sequence was also evaluated (SASQ). Strains expressing the indicated plasmid-encoded Ydj1-CaaX variants and *Sc*FTase (left panels) or *Hs*FTase (right panels) were cultured to equivalent density in liquid media, and 10-fold dilution mixtures spotted onto rich media (YPD) at either room temperature (25°C) or 40°C. The experiment was repeated independently multiple times (*n*=3). (B,C) Gel-shift analysis of Ydj1-CaaX variants. Total cell lysates were prepared from the indicated strains and analyzed as described in Fig. 2D using anti-Ydj1 antibody. See Table S1 for calculations of the percentage of prenylation and replicate numbers. u, unprenylated; p, prenylated. (D) The thermotolerance assay was performed using strains expressing Ydj1 (CASQ) and plasmid-encoded human His_6_-DNAJA2 with either its natural (CAHQ) or an unmodifiable (SAHQ) sequence. The experiment was repeated independently multiple times (*n*=3). (E) Gel-shift analysis of human His_6_-DNAJA2 and His_6_-DNAJA2-SAHQ expressed in wild type (*Sc*), humanized (*Hs*) and FTase-deficient (Δ) yeast. Total cell lysates were prepared from the indicated strains and analyzed as described in Fig. 2D. The upper band for CAHQ did not consistently align with the unprenylated band (see Fig. S3A). Its identity remains obscure, but we speculate that it could be a species with a distinct post-translational modification. The experiment was repeated independently multiple times (*n*=5). u, unprenylated; p, prenylated. The yeast strains used in this figure were: yWS2544 (*Sc*FTase *ydj1*Δ), yWS3186 (*Hs*FTase *ydj1*Δ) and yWS3209 (*ram1*Δ *ydj1*Δ). The plasmids used are listed in Table S4.

To investigate the breadth of sequences recognized by *Hs*FTase, we used a modified version of the humanized FTase yeast system to evaluate a set of well-characterized sequences representing different types of expected modification. For these tests, we evaluated farnesylation of a plasmid-encoded Ydj1-CaaX reporter in a *Hs*FTase *ydj1*Δ strain (Fig. S7). Farnesylation of Ydj1 is required for growth at elevated temperatures (Caplan et al., 1992). Yeast expressing the Ydj1-CaaX variants exhibited growth profiles in the thermotolerance assay that were entirely consistent with previous reports and essentially indistinguishable regardless of whether farnesylation was supported by *Sc*FTase or *Hs*FTase (Fig. 4A) (Hildebrandt et al., 2016b). Ydj1-CASQ and -CAHQ (i.e. shunted sequences) best supported thermotolerance at 40°C; these sequences are derived from yeast Ydj1 and human DNAJA2, respectively. Ydj1-CVIA and -CTLM (i.e. canonical sequences) supported intermediate thermotolerance; these sequences are derived from **a**-factor and Gγ Ste18, respectively. As expected, Ydj1-SASQ (i.e. the unprenylated protein) and a vector control (i.e. no Ydj1) were unable to support growth at high temperature. Although the shunted status of CAHQ has not been verified through biophysical investigations, sequence homology, prediction algorithms, biochemical assays and the above thermotolerance analyses are all consistent with it being a shunted sequence (Hildebrandt et al., 2016b, 2024; Kim et al., 2023). Moreover, CAHQ is resistant to CaaX proteolysis as inferred from mating assays (Fig. S2). Gel-shift analysis of the Ydj1-CaaX variants revealed that both shunted and canonical CaaX sequences were fully prenylated by both *Sc*FTase and *Hs*FTase (Fig. 4B). Additional support for the ability of *Hs*FTase to modify these sequences is evident when comparing the mobilities of Ydj1-CaaX variants in the absence of FTase (i.e. *ram1*Δ) (Fig. 4C).

The observations made for Ydj1 were also evident for human DNAJA2 (CAHQ) that was heterologously expressed in the humanized *Hs*FTase yeast system. DNAJA2 has 48% identity to Ydj1 and can complement some *ydj1*Δ-associated defects (Whitmore et al., 2020). DNAJA2 supported thermotolerance in the context of both *Sc*FTase and *Hs*FTase, whereas DNAJA2-SAHQ did not, indicating that high-temperature growth was prenylation dependent (Fig. 4D). By gel-shift analysis, DNAJA2 was observed to be farnesylated by both *Sc*FTase and *Hs*FTase (Fig. 4E). These results further extend the utility of the humanized *Hs*FTase yeast system for studies of heterologously expressed human CaaX proteins. Moreover, these observations are consistent with the hypothesis that *Sc*FTase and *Hs*FTase have highly similar sequence-recognition profiles that are likely broader than the CaaX consensus sequence.

### *Hs*FTase expressed in yeast modifies a range of human and viral CaaX protein sequences

Studies on the prenylation of CaaX proteins in most systems are hindered by several factors, including difficulty in detecting low abundance proteins, lack of phenotypic readouts, and high labor and time costs for case-by-case studies. Many of these hurdles can be overcome by using the Ydj1 reporter and genetically tractable yeast system described in this study. To extend the utility of our approach, Ydj1-CaaX variants harboring the CaaX sequences of prenylated human and potentially prenylated viral proteins were evaluated by gel-shift assay. Each variant was evaluated in the presence and absence of *Hs*FTase to ensure that gel shifts were due to *Hs*FTase and to assess whether alternate prenylation by *Sc*GGTase-I was possible.

**Fig. 5.**
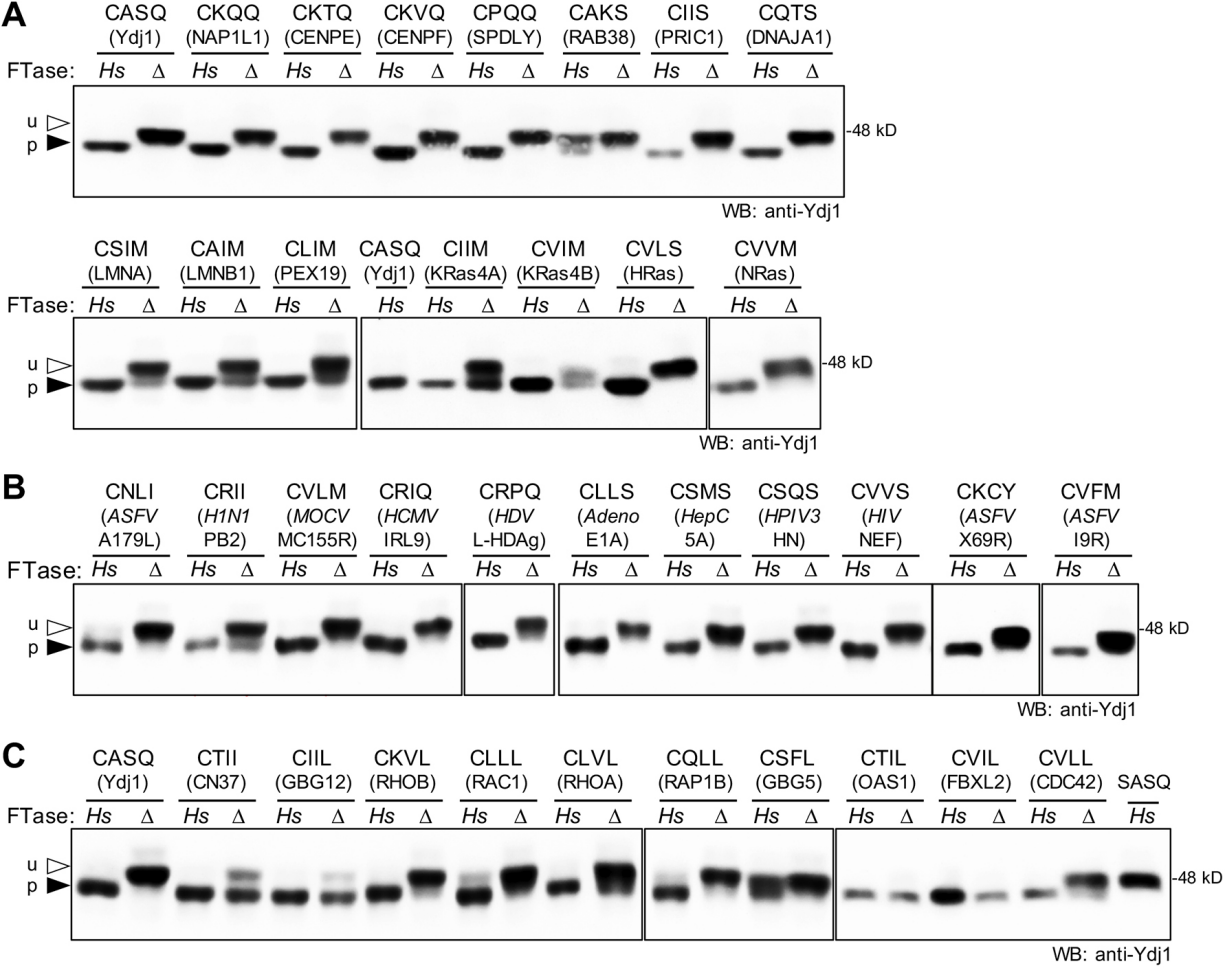
**The humanized *Hs*FTase yeast system modifies Ydj1-CaaX variants derived from human and viral proteins.** The humanized *Hs*FTase yeast system, Ydj1-CaaX reporter and gel-shift analysis were used to evaluate Ydj1-CaaX variants as described in Fig. 2D. The CaaX sequences were derived from (A) human proteins having mostly non-canonical CaaX sequences, (B) mammalian viral proteins and (C) human proteins having mostly CaaL/I sequences. The protein source of the CaaX sequence is indicated below each specific sequence. Virus abbreviations: *H1N1*, human influenza virus; *MOCV*, *Molluscum contagiosum* virus; *HCMV*, human cytomegalovirus; *HDV*, hepatitis delta virus; *Adeno*, human adenovirus 1; *HepC*, human hepatitis C virus; *HPIV3*, human parainfluenza virus 3; *HIV*, human immunodeficiency virus 1; *ASFV*, African swine fever virus. See Table S1 for calculations of the percentage of prenylation and replicate numbers. The strains used in the figure were: yWS3186 (*Hs*) and yWS3209 (Δ). The plasmids used are listed in Table S4. u, unprenylated; p, prenylated.

The Ydj1 reporter system was used to evaluate human CaaX sequences derived from chaperones (Nap1L1, DNAJA1 and Pex19), chromosome stability proteins [CENPE, CENPF and Spindly (SPDL1)], nuclear lamins (prelamin A and lamin B), Ras and Ras-like proteins (Rab38, KRas4A, KRas4B and HRas) and Prickle1 (Fig. 5A) (Ashar et al., 2000; Kho et al., 2004; Kim et al., 1990; Leung et al., 2007; Moudgil et al., 2015; Onono et al., 2010; Palsuledesai et al., 2014; Storck et al., 2019; Strutt et al., 2013; Varela et al., 2008). Apart from the Rab38-associated sequence (CAKS), every sequence was completely modified by *Hs*FTase. CAKS is reported to be underprenylated in its natural protein context (i.e. Rab38), which is consistent with our findings (Köhnke et al., 2013). Sequences ending in methionine (CSIM, CAIM, CLIM, CIIM and CVIM) were fully modified in the presence of *Hs*FTase, yet partially modified in the absence of FTase (*ram1*Δ), suggesting that they are alternately prenylated by *Sc*GGTase-I.

The Ydj1 reporter system was also used to evaluate the prenylation of CaaX sequences associated with human and swine viral proteins (Fig. 5B). The importance of prenylation in viral infection is an emerging line of inquiry for infectious disease therapy, but documentation of viral protein prenylation is limited (Einav and Glenn, 2003; Marakasova et al., 2017). All of the viral CaaX sequences evaluated were modified by *Hs*FTase. These sequences were derived from the influenza virus H1N1 protein PB2 (CRII), *Molluscum contagiosum* virus subtype 1 protein MC155R (CVLM), human cytomegalovirus protein IRL9 (CRIQ), hepatitis delta virus protein L-HDAg (CRPQ), human adenovirus 1 protein E1A (CLLS), hepatitis C virus non-structural protein 5A (NS5A) (CSMS), human parainfluenza virus 3 protein hemagglutinin-neuraminidase (HN) (CSQS), HIV protein NEF (CVVS) and African swine fever virus (ASFV) proteins A179L (homolog of mammalian Bcl-2) (CNLI), X69R (CKCY) and I9R (CVFM). Of these CaaX sequences, only L-HDAg has been biochemically characterized as being prenylated (Glenn et al., 1992). Several others are inferred to be prenylated based on suppression of viral replication by statins (Pronin et al., 2021).

We also evaluated the prenylation of CaaL/I/M sequences derived from well-characterized human CaaX proteins (Fig. 5C). Sequences adhering to this consensus motif are considered targets of GGTase-I or are interchangeably targeted by FTase or GGTase-I (Krzysiak et al., 2010; Trueblood et al., 1993; Zhang et al., 1994). The CaaX sequences were from small GTPases (CDC42, RAC1, RAP1B, RHOA and RHOB), Gγ subunits (GBG5 and GBG12, encoded by *GNG5* and *GNG12*, respectively), the myelin protein CN37 (encoded by *CNP*), the F-box protein FBXL2 and the anti-viral protein Oas1 isoform p46 (De Angelis and Braun, 1994; Katayama et al., 1991; Kawata et al., 1990; Kilpatrick and Hildebrandt, 2007; Onono et al., 2010; Soveg et al., 2021; Storck et al., 2019; Wang et al., 2005; Wickenhagen et al., 2021). All Ydj1-CaaL/I/M variants were fully prenylated in the presence of *Hs*FTase, with the exception of CSFL. Comparing band mobilities in the presence and absence of *Hs*FTase revealed that several sequences were prenylated in a predominantly *Hs*FTase-dependent manner (CKVL, CLLL, CLVL, CQLL, CSFL and CVLL). For the remaining sequences, a mobility shift was still apparent in the absence of *Hs*FTase (CTII, CIIL, CTIL and CVIL), suggesting that these sequences are naturally geranylgeranylated or subject to alternate prenylation in the absence of FTase activity.

### *Hs*FTase modifies sequences with non-canonical lengths

**Fig. 6.**
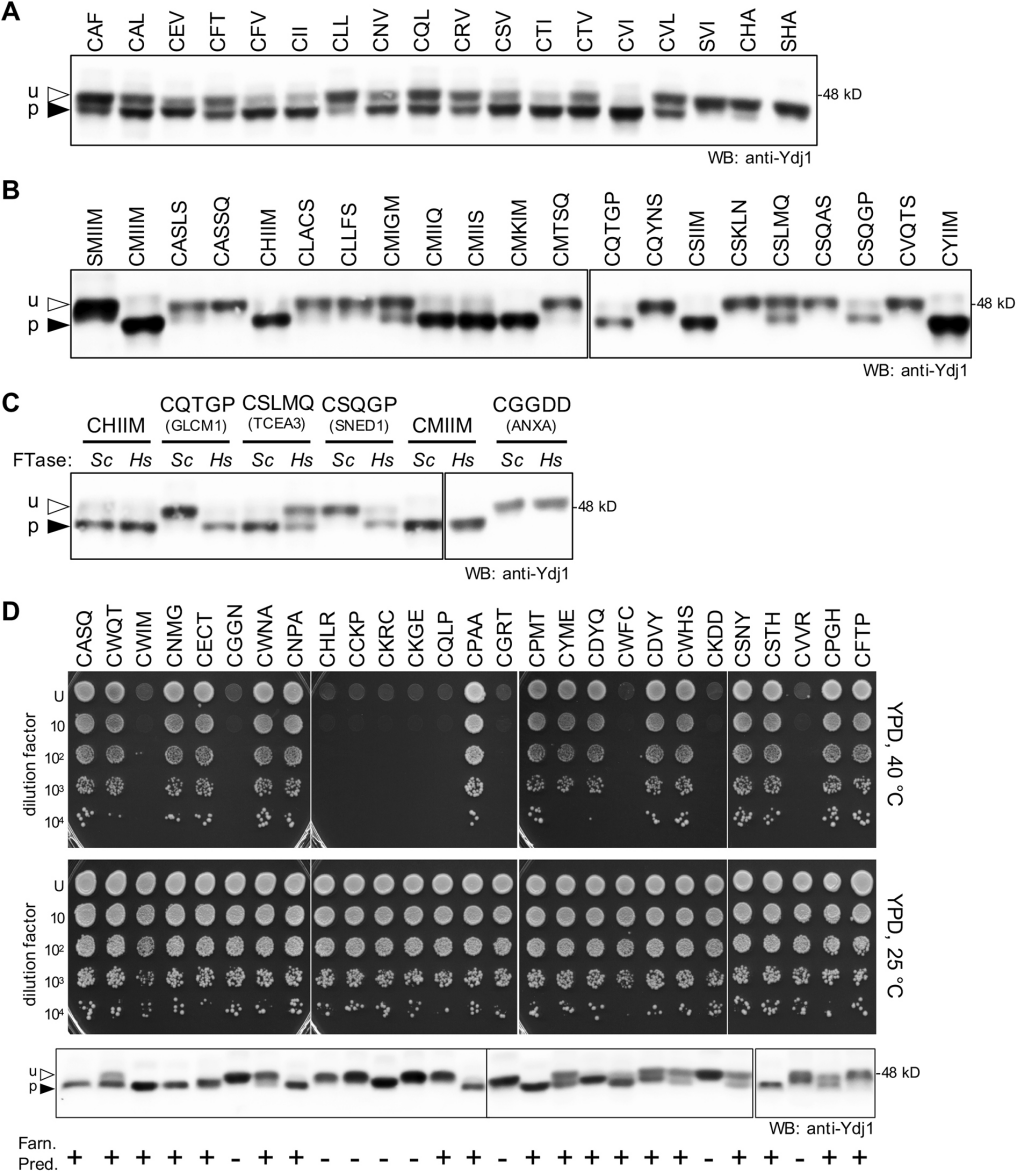
**The humanized *Hs*FTase yeast system modifies CaaX sequences with non-canonical lengths.** (A,B) The humanized *Hs*FTase yeast system, Ydj1-CaaX reporter and gel-shift analysis were used as described in Fig. 2D to evaluate (A) shorter CaX and (B) longer CaaaX sequences. The strain used was yWS3186 (*Hs*FTase *ydj1*Δ). (C) The gel-shift properties of Ydj1-CaaaX variants were compared in the presence of *Sc*FTase (*Sc*) and *Hs*FTase (*Hs*). The protein source of the CaaaX sequence is indicated below each specific sequence where appropriate. The strains used were: yWS3186 (*Hs*FTase ydj1Δ) and yWS2544 (*Sc*FTase *ydj1*Δ). (D) The humanized *Hs*FTase yeast system (yWS3186), thermotolerance assay (upper and middle panels) and gel-shift assay (lower panel) were used to evaluate a random sample of Ydj1-CaaX sequences recovered from a plasmid library encoding all 8000 Ydj1-CaaX variants as described in Fig. 2C,D. Predictions for farnesylation of each sequence are indicated on the bottom. Predictions were derived using heat map (HM) scores (Kim et al., 2023): positive prediction, HM score >3; negative prediction, HM score <3. For A-C, the plasmids used are listed in Table S4. See Table S1 for calculations of the percentage of prenylation and replicate numbers. u, unprenylated; p, prenylated.

The substrate recognition profile of yeast FTase has recently been expanded to certain sequences one amino acid longer or shorter than the tetrapeptide CaaX sequence (i.e. CaX and CaaaX). This expanded specificity was first identified using a combination of yeast genetics and *in vitro* biochemical analyses using synthetic peptides and purified FTase (Ashok et al., 2020; Blanden et al., 2018; Schey et al., 2021; 2024). To extend these observations to *Hs*FTase, previously studied Ydj1-CaX and -CaaaX variants were expressed in the humanized *Hs*FTase yeast system and analyzed by gel-shift assay (Fig. 6). One additional CaX (CHA) sequence was evaluated due to its association with the SARS-CoV-2 protein ORF7b.

The 16 shorter CaX sequences evaluated exhibited varying degrees of farnesylation ranging from >90% (CVI and CTI) to <25% (CLL and CHA), with half exhibiting >50% farnesylation (eight of 16) (Fig. 6A; Table S1). The farnesylation patterns were similar to those previously observed for *Sc*FTase (Ashok et al., 2020). The very weak farnesylation seen for Ydj1-CHA, which was quantified to be ∼8% of the population, was not evident for Ydj1-SHA, consistent with the cysteine-dependent nature of prenylation, but it remains to be determined whether such a low level of prenylation is biologically significant for the biology of SARS-CoV-2.

The 20 longer CaaaX sequences evaluated also exhibited varying degrees of farnesylation ranging from >90% farnesylation (e.g. CMIIM) to no farnesylation (e.g. CASSQ), with nearly half exhibiting >50% farnesylation (nine of 20) (Fig. 6B). Most sequences were similarly modified by *Sc*FTase and *Hs*FTase. Some partially modified sequences exhibiting varied prenylation (Table S1). Of note, the CGGDD sequence (annexin A2, encoded by *ANXA2*) was unmodified in the humanized *Hs*FTase yeast system, despite being identified as farnesylated through methods involving metabolic labeling with an azido-farnesyl analog (Kho et al., 2004). Among the most dramatic differences in farnesylation by the yeast and human FTases were seen for CQTGP (GLCM1, encoded by *GLYCAM1*) and CSQGP (SNED1), which were modified by *Hs*FTase but not by *Sc*FTase, and CSLMQ (TCEA3), which was fully modified by *Sc*FTase but <50% was modified by *Hs*FTase (Fig. 6C). The differences in species specificity observed for these and other CaaaX sequences indicate differences in the substrate-binding pockets of these enzymes, which warrants further investigation.

This survey of CaX and CaaaX sequences reveals that *Hs*FTase can indeed modify sequences with non-canonical lengths in a cell-based system. For many of the sequences associated with eukaryotic or viral proteins (see Table S2), it remains to be determined, however, whether they are prenylated in their native context.

### The humanized FTase yeast strain can be used to identify *Hs*FTase target sequences

Yeast genetic screens using HRas and Ydj1 reporters have comprehensively evaluated all 8000 CaaX sequences for reactivity with *Sc*FTase (Kim et al., 2023; Stein et al., 2015). These genetic strategies are fully compatible with the humanized system developed in this study that could be used to query CaaX sequence space recognized by *Hs*FTase. As proof of principle, we sampled a Ydj1-CaaX plasmid library using the humanized *Hs*FTase system along with thermotolerance and gel-shift assays (Fig. 6D; Table S1). The results for a randomly sampled set of 26 Ydj1-CaaX variants revealed that farnesylation of Ydj1-CaaX by *Hs*FTase correlated with thermotolerant growth properties. Exceptions were Ydj1-CWIM and -CWFC, which were farnesylated as judged by gel shift yet were temperature sensitive. The observations for CWIM and CWFC are consistent with the profile of canonical CaaX sequences (i.e. fully-modified: farnesylated and cleaved, as well as carboxyl methylated at the end of the protein sequence) that temper the ability of Ydj1 to support thermotolerance (Berger et al., 2018; Hildebrandt et al., 2016b).

Studies of FTase specificity have led to the development of predictive models for prenylation (Berger et al., 2022; Kim et al., 2023). The more recent of these models based on *Sc*FTase was used to predict the farnesylation (positive and negative) of the randomly sampled Ydj1-CaaX variants (Fig. 6D; see bottom of the panel). The predictions correlated well with empirical observations for positive and negative modification by *Hs*FTase, with a majority of sequences (20 of 26) being accurately predicted using rigorous thresholds (>50% prenylation as determined by band quantitation was required for positive classification; <10% prenylation was required for negative classification). For classification purposes, CKRC was judged to be unmodified because the gel mobility associated with this sequence also occurred in the absence of FTase (Fig. S3B). Of the outlier sequences, some were predicted to be modified but exhibited prenylation below the 50% threshold for positive classification (i.e. CWNA, CWHS, CSNY and CPGH). By contrast, two outlier sequences that were predicted to be prenylated were unmodified (i.e. CQLP and CFTP), suggesting differences in specificity between yeast and human FTase. Overall, the *Sc*FTase-based prediction model suggested ∼75% accuracy for predicting prenylation of a CaaX sequence by *Hs*FTase (Kim et al., 2023).

An obvious caveat of any predictive model is that the sequence must be accessible in its native context for modification by cytosolic FTase. Although many sequences tested in this study are associated with known prenylated proteins, others that exist on human proteins have not been investigated. For example, querying the UniProtKB/Swiss-Prot database for sequences from the limited-scale genetic screen revealed that CGGN and CKRC exist on human proteins (Table S2), but our data indicate that they cannot be farnesylated (Fig. 6D). We thus predict that they will not be farnesylated in their native contexts. CPAA, which is associated with the human endoplasmic reticulum-localized DNase-1-like 1 protein (DNASE1L1) and the extracellular laminin subunit α4 (LAMA4), was modified by *Hs*FTase. The subcellular localizations of these human proteins likely disqualify them from being substrates of cytosolic FTase. Nevertheless, the observation that the substrate specificities of *Sc*FTase and *Hs*FTase are highly similar but not identical prompts the need for future studies aimed at fully evaluating CaaX sequence space in the context of *Hs*FTase. Such studies are now possible with our humanized FTase yeast system, and such investigations may better refine the target profile of *Hs*FTase, potentially leading to the discovery of previously unreported CaaX proteins.

### Interspecies complementation analysis of GGTase-I subunits

**Fig. 7.**
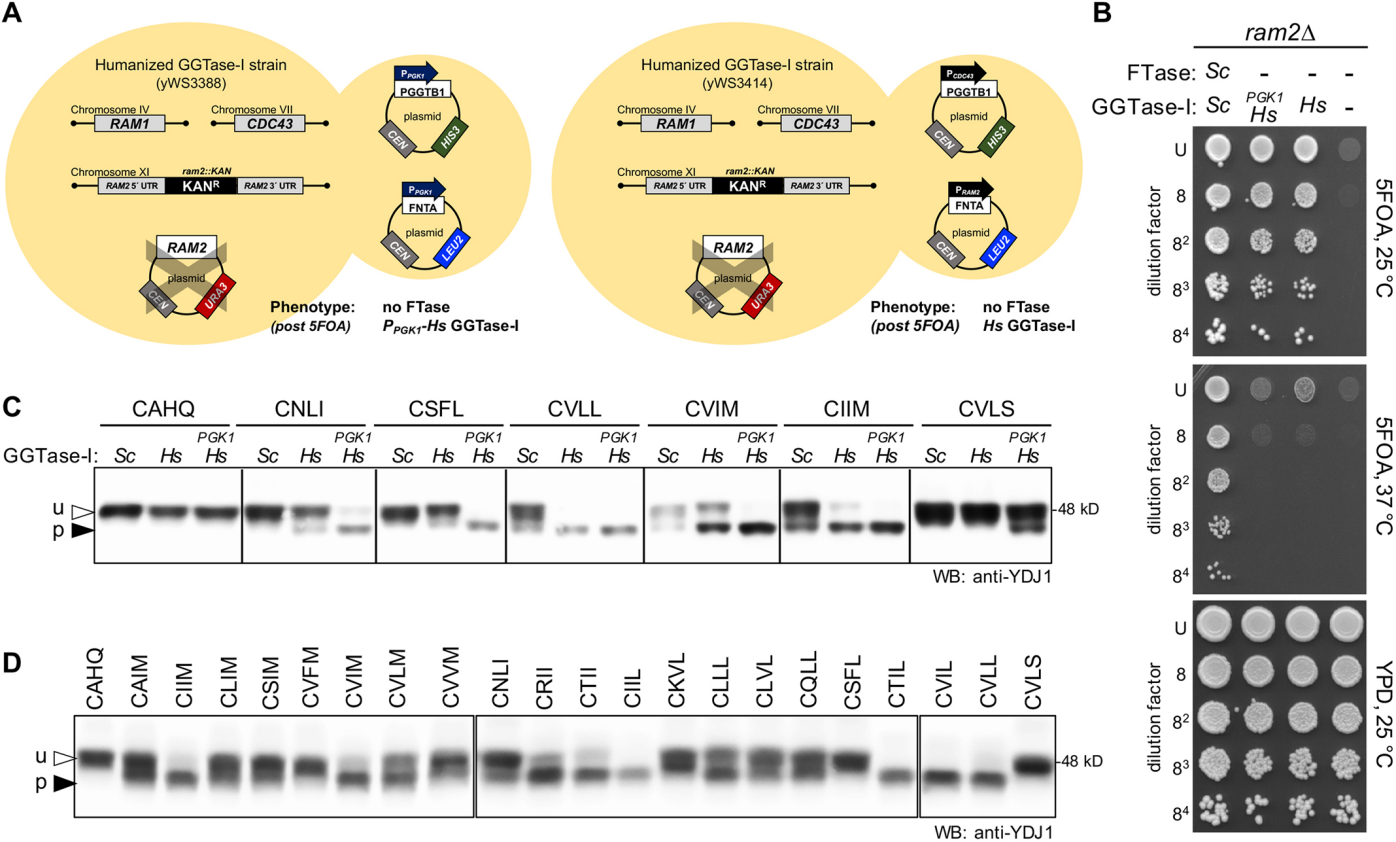
**The humanized *Hs*GGTase-I yeast system geranylgeranylates CxxL/I/M sequences.** (A) Key genetic features of the humanized *Hs*GGTase-I strains. Coding sequences for human GGTase-I subunits were expressed in a *ram2*Δ genetic background. yWS3414 contains plasmids encoding *Hs*FNTA(α) and *Hs*PGGT1B(β) driven by orthologous yeast gene promoters (*Hs*); yWS3388 has both genes driven by the *PGK1* promoter (*_PGK1_Hs*). The semi-transparent ‘X’ overlays the plasmid encoding *RAM2*(α) that is absent from the strain after 5FOA counter-selection. (B) The humanized *Hs*GGTase-I strains described in panel A, along with positive and negative controls, were evaluated for GGTase-I activity using the 5FOA assay. Strains were cultured to the equivalent density in selective liquid media and serially diluted, and the dilution series mixtures were replica spotted onto 5FOA and YPD solid media. 5FOA counter-selects for the *URA3*-marked plasmid, making growth reliant on genes encoded on the remaining *HIS3*- and *LEU2*-marked plasmids. Growth on 5FOA at 37°C requires FTase activity, which is absent in the humanized *Hs*GGTase-I strains. Growth on YPD provides an assessment for the quality of the serial dilutions. The strains used were: yWS3388, yWS3411, yWS3414 and yWS3481. The experiment was repeated independently multiple times (*n*=5). (C,D) Gel-shift analysis of Ydj1-CaaX variants produced in yeast expressing *Sc*GGTase-I (*Sc*) or plasmid-encoded *Hs*GGTase-I (*Hs* or *_PGK1_Hs*). The CaaX sequences were a mix of canonical and non-canonical sequences (C) or CaaM/I/L sequences derived from human proteins (D). Total cell lysates were prepared and analyzed as described in Fig. 2D. The strains used were: yWS3209 (*Sc*), yWS3451 (*Hs*) and yWS3169 (*_PGK1_Hs*); yWS3451 was exclusively used in panel D. The plasmids used are listed in Table S4. See Table S1 for calculations of the percentage of prenylation and number of replicates.

To develop a yeast system for expressing *Hs*GGTase-I, studies were also performed to investigate the functional equivalence of *Sc*GGTase-I and *Hs*GGTase-I subunits. Because GGTase-I activity is essential for yeast viability, strains lacking either subunit of *Sc*GGTase-I can only be propagated when complemented by a plasmid-encoded copy of the missing gene. Pertinent yeast strains were created and, in a manner similar to FTase studies, they were transformed with plasmid-encoded copies of both orthologous *Hs*GGTase-I subunit genes *Hs*FNTA(α) and *Hs*PGGT1B(β) under different promoter conditions (Fig. 7A). The strains were evaluated using a growth assay [i.e. 5-fluroorotic acid (5FOA) assay] that counter-selects for the *URA3*-marked plasmid encoding the *Sc*FTase*/*GGTase-I α subunit gene (i.e. *RAM2*), resulting in yeast that lack FTase and are reliant on *Hs*GGTase-I for growth. Regardless of whether subunits were expressed from the orthologous yeast promoters or the *PGK1* promoter, strains producing *Hs*GGTase-I exhibited robust growth nearly equivalent to yeast expressing endogenous *Sc*GGTase-I (Fig. 7B). As expected, yeast strains lacking *RAM2* did not support growth on 5FOA at 37°C because they lack FTase activity.

The 5FOA assay also revealed, as observed for FTase, that neither *Hs*FNTA(α) nor *Hs*PGGT1B(β) alone could complement for the absence of their yeast orthologs *Sc*Ram2(α) and *Sc*Cdc43(β), respectively, indicating that interspecies subunits do not form functional GGTase-I complexes (Fig. S4A). The results did not change when human subunits were overexpressed using the constitutive *PGK1* promoter. This observation contrasts with the situation in which both *Hs*FTase subunits had to be expressed from the *PGK1* promoter for functional complementation. The reason for this discrepancy is unknown but might reflect differences in promoter strength (*P_RAM1_* versus *P_CDC43_*) or β subunit stability.

### *Hs*GGTase-I expressed in yeast modifies both canonical and non-canonical CaaX sequences

Evidence suggests that GGTase-I has broader than anticipated substrate specificity at the a_1_ and a_2_ positions than implied by the CaaL/I/M consensus sequence (Kawata et al., 1990; Kilpatrick and Hildebrandt, 2007; Lebowitz et al., 1997; Onono et al., 2010; Sarkar et al., 2024). We investigated this issue using the humanized *Hs*GGTase-I yeast system, plasmid-encoded Ydj1-CaaX variants, and gel-shift analysis. The yeast strains expressed either endogenous *Sc*GGTase-I or *Hs*GGTase-I for which subunit expression was driven by either orthologous *RAM2*(α) and *CDC43*(β) promoters (*Hs*) or the constitutive *PGK1* promoter (*_PGK1_Hs*) (Fig. S7). This analysis revealed that neither GGTase-I could modify Ydj1-CAHQ, a sequence associated with DNAJA2 that is strictly farnesylated (Andres et al., 1997). A mix of canonical (CVLL, CVIM, CIIM and CVLS) and non-canonical (CNLI and CSFL) CaaX sequences exhibited no or limited prenylation by *Sc*GGTase-I and each was better modified by *Hs*GGTase-I (Fig. 7C). This was more evident when *Hs*GGTase-I subunits were expressed using the *PGK1* promoter, presumably due to increased protein levels of *Hs*GGTase-I. Collectively, these observations indicate that *Hs*GGTase-I has the potential to modify certain non-canonical CaaL/I/M sequences (e.g. CNLI and CSFL) and that *Hs*GGTase-I expressed in the yeast system has more activity and/or different specificity than that of *Sc*GGTase-I.

To further survey the geranylgeranylation potential of CaaX sequences, we evaluated a test set of 22 sequences similar to the CaaL/I/M consensus using the humanized *Hs*GGTase-I yeast system with lower GGTase-I activity (i.e. the system in which expression is driven using orthologous gene promoters). This was done to reduce the potential of overexpression artifacts. Most sequences exhibited a gel shift, but only a few were fully shifted, indicative of complete (i.e. CIIL, CTIL, CVIL and CVLL) or near-complete (CIIM, CVIM and CTII) geranylgeranylation (Fig. 7D). Within this data set, we observed that the a_1_ position influenced prenylation by *Hs*GGTase-I. This is evident when comparing the CxIM set of sequences: CSIM, CAIM and CLIM (∼50% modified) versus CIIM and CVIM (100% modified). A similar observation was made when comparing CxLL sequences: CLLL and CQLL (∼50% modified) versus CVLL (100% modified). Sequences in which the X position did not match that in the CaaL/I/M consensus (CAHQ, CVLS) were unmodified by *Hs*GGTase-I. Lastly, we observed that GFP-Ras2-CIIM (KRas4A) and GFP-Ras2-CVIM (KRas4B) were prenylated by *Hs*GGTase-I, as inferred from previous observations (see Fig. 3 and Fig. S4B).

### Human lamins and Pex19 CaaX sequences have distinct CaaX protease profiles

The ability to humanize yeast for investigations of prenyltransferase activities can be adapted to studies of the CaaX proteases that are gatekeepers for the canonical CaaX modification pathway. Rce1 cleaves certain prenylated CaaX proteins, allowing for carboxymethylation. Its activity is prenyl dependent and target sequences typically have an aliphatic amino acid at a_2_ (Kim et al., 2023; Plummer et al., 2006). Ste24 (also known as ZMPSte24) has also been identified as a CaaX protease, despite a lack of widespread CaaX protein substrates and an ability to cleave non-prenylated sequences (Boyartchuk et al., 1997; Hildebrandt et al., 2016a). Ste24 has an unrelated and likely primary function in protein quality control (Ast et al., 2016; Runnebohm et al., 2020).

**Fig. 8.**
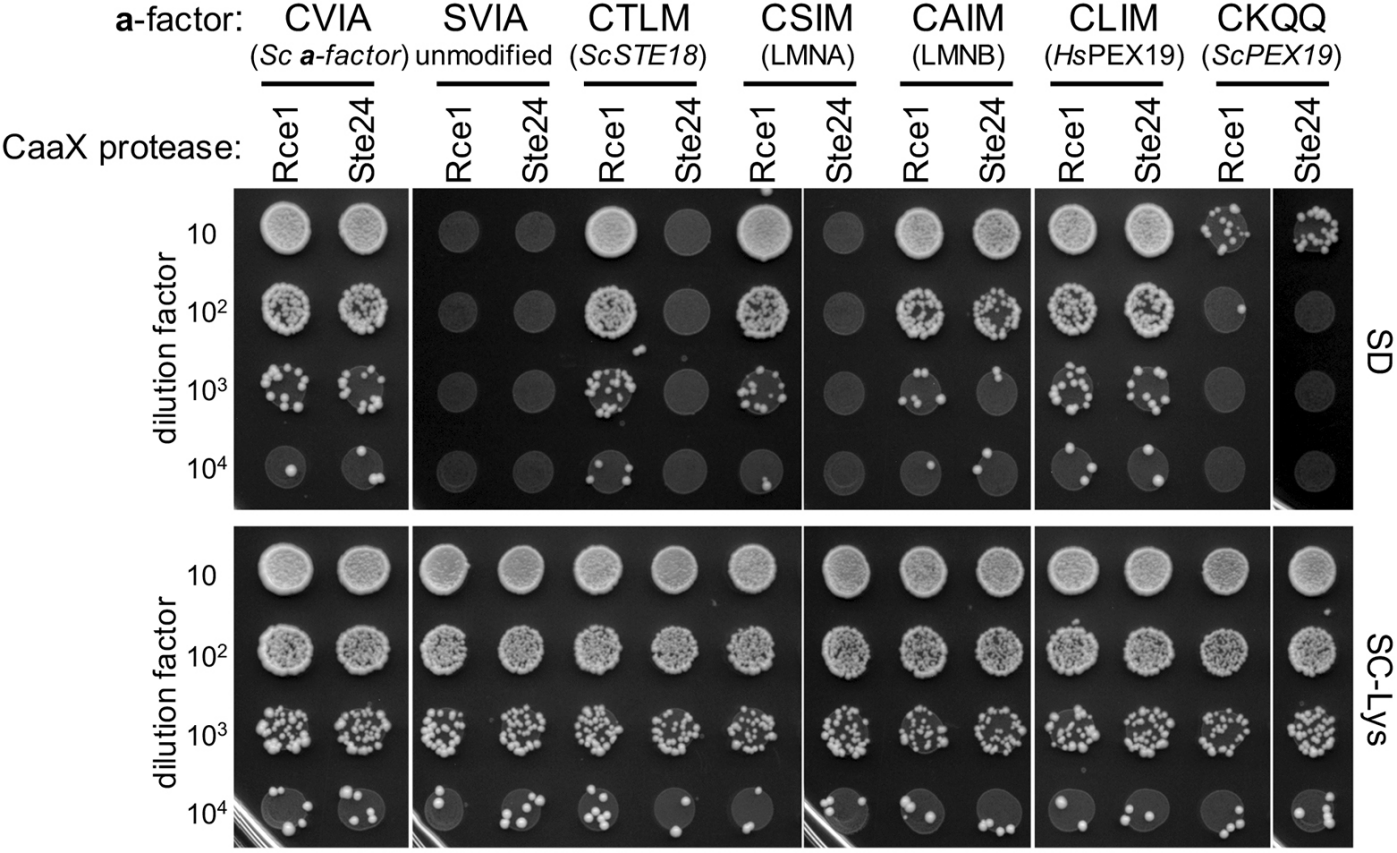
**Prenylation studies can be coupled with CaaX proteolysis studies to better understand CaaX modifications.** The **a**-factor-dependent mating assay was performed as described in Fig. 2B using *MAT***a** strains carrying plasmids encoding **a**-factor-CaaX variants and either the *Hs*Rce1 (Rce1) or ZMPSTE24 (Ste24) CaaX protease. The protein source of the CaaX sequence is indicated below each specific sequence. The strain background used lacks endogenous **a**-factor and yeast CaaX protease genes (yWS164; *MAT***a** *mfa1*Δ *mfa2*Δ *rce1*Δ *ste24*Δ). The plasmids used are listed in Table S4. The experiment was repeated independently multiple times (*n*=3).

The yeast mating assay (i.e. **a**-factor production) has been used to evaluate the specificities of heterologously expressed human Rce1 and ZMPSte24 (Mokry et al., 2009; Plummer et al., 2006). Studies of CaaX protease specificity are critical for resolving the cleavage preferences of medically relevant human proteins. For example, prelamin A (CSIM), which is associated with progeria-like diseases, is subject to two proteolytic events: one within the CaaX sequence and another 15 amino acids from the C-terminus (Barrowman et al., 2012b). Both cleavage events are needed to produce lamin A and have been attributed to ZMPSte24 (Barrowman et al., 2012a; Quigley et al., 2013). With the humanized CaaX protease yeast system, we observed that yeast mating, indicative of **a**-factor CaaX cleavage, occurred with **a**-factor-CSIM when *Hs*Rce1 was present, and not with ZMPSte24, in accordance with more recent observations (Fig. 8) (Berger et al., 2022; Nie et al., 2020 preprint). We extended these studies to evaluate the lamin B CaaX sequence (CAIM), which led to a mating phenotype with *Hs*RCE1 or ZMPSte24. We also examined peroxisomal chaperone Pex19 sequences. Farnesylation of Pex19 is critical for its interaction with client proteins, and mutations of human Pex19 unrelated to its CaaX sequence are associated with Zellweger syndrome (Emmanouilidis et al., 2017; Matsuzono et al., 1999). Human Pex19 (CLIM) and yeast Pex19 (CKQQ) have strikingly different CaaX sequences. Both were fully prenylated by yeast and human FTases (see Fig. 5A and Fig. S5A). With the humanized CaaX protease system, we observed mating for **a**-factor-CLIM with *Hs*RCE1 or ZMPSte24, whereas no mating was observed with **a**-factor-CKQQ. This result suggests that Pex19 proteins have, for reasons unknown, evolved C-termini with distinct biophysical properties. Taken together, our studies fully support the combined utility of the humanized prenyltransferase and CaaX protease yeast systems for profiling the CaaX modification potential of different sequences. Such studies could prove useful for predicting the potential effects of inhibiting the Rce1 CaaX protease, which would impact a narrower range of CaaX protein targets than prenyltransferase inhibitors.

## DISCUSSION

We report the development of yeast systems useful for systematic characterization of prenylation and cleavage of CaaX sequences by human prenyltransferases and CaaX proteases. The humanized *Hs*FTase system, in particular, was phenotypically equivalent to yeast expressing native *Sc*FTase in various assays: (1) **a**-factor-dependent mating, (2) Ydj1-based thermotolerance, (3) GFP-Ras2 localization and (4) gel shift of distinct reporters. The humanized *Hs*FTase yeast system is an accessible and rapid tool for characterizing the farnesylation potential of candidate CaaX sequences and heterologously expressed human CaaX proteins. Importantly, this system is cell based, which alleviates concerns associated with *in vitro* farnesylation assays that depend on chemically modified substrates, and unnatural amounts of enzyme and substrate. A limitation of this system, however, is that CaaX sequences determined to be prenylated using any of our reporters (i.e. Ydj1, Ras2 and **a**-factor) will still need to be validated in their native context. For example, the accessibility of a CaaX sequence and the properties of sequences upstream of CaaX may constrain reactivity (i.e. the subcellular localization of the substrate, buried C-terminus, etc.) (Bond et al., 2017; Hicks et al., 2005; Reid et al., 2004). Thus, any positive results with our system can should be coupled with additional studies. Nonetheless, the ease and convenience of the humanized *Hs*FTase yeast system that we have developed is an important and valuable new resource for initial and supporting investigations on the farnesylation potential of CaaX sequences.

Limited comparative data exists for the specificities of mammalian FTase relative to that of *Sc*FTase. *In vitro*, *Sc*FTase and rat FTase exhibit similar specificities when evaluated against a CVa_2_X peptide library (Wang et al., 2014). Our *in vivo* results confirm and further extend such observations to reveal that *Sc*FTase and yeast-expressed *Hs*FTase have a striking degree of substrate conservation (see Figs 2, 4 and 5, Fig. S5 and Table S1). This is most evident in the ability of both enzymes to similarly farnesylate canonical and non-canonical sequences (i.e. sequences lacking aliphatic amino acids at a_1_ and a_2_). These findings indicate that farnesylation-prediction algorithms developed using *Sc*FTase should work reasonably well for predicting *Hs*FTase specificity (Kim et al., 2023). The conservation between human and yeast FTases extends to prenylation of sequences with non-canonical lengths (i.e. CaX and CaaaX), although species-specific differences in substrate recognition of longer sequences were observed. Toward mechanistically understanding such differences, it will be important to compare high-resolution structures of mammalian and yeast FTase, which have limited primary sequence identity (22-34%). Although many mammalian X-ray crystal structures of FTase exist, there is currently no *Sc*FTase structure. Overlay of the *Hs*FTase crystal structure and yeast FTase structural models predicted by AlphaFold, however, revealed excellent conservation of residues that coordinate the a_2_ and X positions of CaaX sequences (Fig. S6), consistent with our observations that these enzymes have highly similar substrate specificities.

This study also produced a humanized *Hs*GGTase-I yeast system for characterizing the geranylgeranylation potential of candidate CaaX sequences that, presumably, can be used for analyses of heterologously expressed human CaaX proteins. Human GGTase-I prenylated a wide range of CaaL/I/M sequences but did not prenylate CaaS/Q sequences, in alignment with expectations. Both yeast and human GGTase-I had the ability to alternately prenylate similar sequences in the context of GFP-Ras2 (i.e. CIIM and CVIM) and Ydj1 (i.e. CAIM, CIIM, CKVL, CLIM, CLLL, CLVL, CSIM and CVIM) (Figs 3, 5 and 7; Fig. S4B; Table S1). This typically occurs when FTase activity is reduced through action of farnesyltransferase inhibitors or, in our case, when FTase activity was genetically ablated. This observation demonstrates that recognition of alternately prenylated sequences by GGTase-I is a transferable property (i.e. it occurs in other protein contexts) and is evolutionarily conserved between yeast and human enzymes, and likely among other species as well. A caveat of the humanized *Hs*GGTase-I yeast system, however, is that it displayed more robust activity than that of endogenous yeast GGTase-I in modifying Ydj1-CaaL/I/M variants. This observation could be due to higher *Hs*GGTase-I enzyme levels and/or specificity differences between the yeast and human enzymes. An overlay of the rat GGTase-I crystal structure and yeast GGTase-I AlphaFold models predicts different active site architectures for these enzymes, which could explain our observations (Fig. S6).

The scope of potentially prenylated proteins in protein databases is expansive, with over 6000 annotated proteins in eukaryotes and viruses ending in Cxxx. When expanded to 3-mer and 5-mer length sequences, this number is over 15,000 (Fig. 1C). Not accounted for in this analysis is the potential number of prokaryotic CaaX proteins. Although prokaryotes lack protein prenylation machinery, certain pathogenic bacteria (e.g. *Legionella pneumophila* and *Salmonella typhimurium*) encode CaaX proteins that are prenylated by host enzymes as part of their infectious life cycle (Ivanov et al., 2010; Reinicke et al., 2005). Thus, reliable prediction algorithms and reporter systems will be helpful in identifying and verifying the subpopulation of CaaX sequences modified by *Hs*FTase or *Hs*GGTase-I against the backdrop of infectious disease.

Viral genomes alone encode many under-characterized proteins that might be prenylated by host enzymes involved in CaaX protein modification. The viral-derived CaaX sequences tested in this study were all farnesylated. We observed that CRPQ (hepatitis delta virus large delta antigen, L-HDAg) is modified by both human and yeast FTases, but not by GGTase-I. Furthermore, we observed that the CRPQ sequence is not cleaved, consistent with its non-canonical nature (Fig. 5; Fig. S2). Farnesylation of L-HDAg is required for virion assembly, and a farnesyltransferase inhibitor (lonafarnib) is currently in phase 3 clinical trials for use in combination treatments for hepatitis D infections (Khalfi et al., 2023; Koh et al., 2015; Yardeni et al., 2022). We also determined that CRIQ (cytomegalovirus protein of unknown function IRL9) can be farnesylated, cleaved and carboxymethylated. CaaX modification of IRL9 has not been previously reported, but it is a good candidate for additional investigations as statins that block production of mevalonate (a precursor of farnesyl and geranylgeranyl groups) are known to diminish cytomegalovirus replication (Potena et al., 2004). Our yeast systems revealed that other viral CaaX sequences, i.e. CRII (influenza virus H1N1 protein PB2), CVLM (*M. contagiosum* protein MC155R) and CNLI (ASFV protein A179L), were substrates of both *Hs*FTase and *Hs*GGTase-I. If these sequences are prenylated in their native contexts, dual prenyltransferase inhibitors might have better utility for blocking their prenylation. Given that all the viral-derived CaaX sequences evaluated in our study were prenylated, it is prudent to consider the role that the prenylation of viral proteins may have on viral propagation and infection.

In summary, we developed three humanized yeast systems useful for probing the modification potential of CaaX sequences. These systems provide an accessible and rapid avenue for determining whether a specific CaaX sequence is unmodified, prenylated (i.e. farnesylated or geranylgeranylated) or additionally cleaved. Such investigations are critical first steps for characterizing any CaaX protein for which the modification status is unknown. We expect that individual CaaX sequences determined to be unmodified by our systems will likely be unmodified in their native protein contexts, whereas modified sequences will need to be fully vetted in their natural contexts to confirm predictions derived using these systems. Additionally, the data collected in this study suggest that yeast and human FTase specificities are largely preserved, consistent with their conserved active site architectures, whereas yeast and mammalian GGTase-I specificities are less preserved, consistent with proposed differences in their active site architectures.

## MATERIALS AND METHODS

### Yeast strains

The manipulated genomes of humanized prenyltransferase strains are summarized in Fig. S7. Detailed genotypes are listed in Table S3, with detailed strain construction strategies given in the supplementary Materials and Methods. Yeast were typically cultured in synthetic complete (SC) or yeast extract-peptone-dextrose (YPD) liquid and solid yeast media unless otherwise noted. For expression of Myc-HRas^Q61L^ from the inducible *MET25* promoter (p-05547) in methionine auxotroph strains (i.e. yWS2544 and yWS3186), yeast were cultured in SC-leucine containing 20 µg/ml methionine to late log phase, washed and resuspended in SC-leucine containing 2 µg/ml methionine to allow for both growth and induction of the *MET25* promoter.

New strains were created for this study by standard genetic manipulations starting with commercially available haploid and heterozygous diploid genomic deletions. *KAN^R^*- and *NAT^R^*-marked gene replacements were confirmed by growth on YPD medium containing G418 (200 µg/ml; Research Products International) or nourseothricin (100 µg/ml; GoldBio), respectively. All gene replacements were further checked by PCR to confirm the presence of the knockout at the correct locus and absence of the wildtype open reading frame (ORF). Plasmids were introduced into strains via a lithium acetate-based transformation procedure (Elble, 1992). Yeast sporulation was carried out in a solution of 2% potassium acetate, 0.25% yeast extract and 0.1% dextrose. Spore enrichment and random spore analysis followed published methods (Rockmill et al., 1991). For the humanized FTase strains (yWS3186 and yWS3220), *P_PGK1_-FNTB* was integrated at the *RAM1* locus, replacing the ORF, using a loop-in-loop-out strategy, and *P_PGK1_-FNTA* was integrated at the *his3*Δ*1* locus by homologous recombination using a *HIS3*-based integrative plasmid.

### Plasmids

A comprehensive list of plasmids is given in Table S4 and detailed plasmid construction strategies are in the supplementary Materials and Methods. Plasmids were routinely analyzed by diagnostic restriction digestion and DNA sequencing (Eurofins Genomics, Louisville, KY, USA) to verify the entire ORF and surrounding sequence. Plasmids recovered from the Ydj1-CaaX Trimer20 library are described elsewhere (Kim et al., 2023). New plasmids encoding Ydj1-CaaX and **a**-factor-CaaX (encoded by *MFA1*) variants were made by PCR-directed plasmid-based recombinational cloning as previously described (Berger et al., 2018; Oldenburg et al., 1997). Plasmids with GFP-Ras2-CaaX variants were generated using a modified QuikChange site-directed mutagenesis procedure; complementary mutagenic primers were used for whole plasmid PCR of pWS1735 using Q5 polymerase (New England Biolabs) and an 8-min extension time, with products being digested with *DpnI* (New England Biolobs) prior to *E. coli* transformation. Plasmids encoding *Hs*FTase and *Hs*GGTase-I subunits were created in multiple steps. First, the ORFs and flanking 5′ and 3′ sequences of yeast prenyltransferase subunits were PCR-amplified from strain BY4741 and subcloned into the multicloning sites of appropriate pRS series vectors (Sikorski and Hieter, 1989). In parallel, synthetic cDNAs for human *FNTA*, *FNTB* and *PGGT1B* that were codon optimized for *S. cerevisiae* were subcloned into the PstI and XhoI sites of pBluescriptII KS(-); all cDNAs were commercially obtained (GenScript). The human ORFs were engineered to also have 39 bp of flanking sequence on both the 5′ and 3′ends, which equivalently matched the flanking sequences of the orthologous yeast ORFs. Next, DNA fragments encoding the human ORFs and yeast flanking sequences were used with recombination-based methods for direct gene replacement of the plasmid-encoded yeast ORFs. The *PGK1* promoter was amplified and used to replace the orthologous yeast promoters by similar recombination-based methods. To change selectable markers, the various FTase and GGTase-I promoter-containing segments were subcloned as XhoI-SacI fragments into different pRS vector backbones by conventional ligation-based cloning.

### Thermotolerance assay

The assay was performed as previously described (Blanden et al., 2018; Hildebrandt et al., 2016b). In brief, plasmid-transformed strains were cultured in appropriate selective synthetic drop-out (SC−) liquid media to saturation (25°C, 24 h); strains without plasmids were cultured in SC complete media. Cultures were serially diluted into H_2_O (10-fold dilutions), and dilution mixtures replica spotted onto YPD plates. Plates were incubated (25°C for 72 h; 37°C for 48 h; 40°C and 41°C for 72 h) then digitally imaged using a Canon flat-bed scanner (300 dpi; grayscale; TIFF format).

### Yeast lysate preparations and immunoblotting

Cell-mass equivalents of log-phase yeast [absorbance at 600 nm (A_600_) of 0.95-1.1) cultured in appropriate selective SC− liquid media were harvested by centrifugation, washed with water, and processed by alkaline hydrolysis and trichloroacetic acid precipitation (Kim et al., 2005). Total protein precipitates were resuspended in urea-containing sample buffer (250 mM Tris, 6 M urea, 5% β-mercaptoethanol, 4% SDS, 0.01% bromophenol blue, pH 8) and analyzed by SDS-PAGE (9.5%), followed by immunoblotting. Blots were processed according to standard protocols. Antibodies used for western blots were: rabbit anti-Ydj1 polyclonal (1:10,000; gift from Avrom Caplan, City College of New York); mouse anti-c-Myc 9E10 monoclonal (1:1000, Santa Cruz Biotechnology, sc-40); mouse anti-GFP B-2 monoclonal (1:500, Santa Cruz Biotechnology, sc-9996); mouse anti-DnaJA2(7) monoclonal (1:500, Santa Cruz Biotechnology, sc-136515); mouse anti-*Sc*Ras2 monoclonal (1:600, Santa Cruz Biotechnology, sc-365773); mouse anti-HIS his.h8 monoclonal (1:1000, VWR, 101981-852); and goat HRP-anti-rabbit and goat HRP-anti-mouse (1:1000, Kindle Biosciences, R1006 and R1005, respectively) antibodies. Immunoblots were developed with ECL reagent (ProSignal Pico ECL Spray or Kindle Biosciences KwikQuant Western Blot Detection Kit) and images were digitally captured using the KwikQuant Imager system (Kindle Biosciences). Adobe Photoshop was used for cropping and rotating of images; no other image adjustments were applied.

### Quantification of prenylation from immunoblot images

Prenylation of Ydj1-CaaX was quantified using ImageJ and at least two exposures of each digital image having good dynamic range of band intensities. The percentage of prenylation was calculated as the intensity of the lower (prenylated) band divided by the total intensity of the prenylated and unprenylated (upper) bands. Data presented in Table S1 represent the averages of the indicated number of biological replicates. For duplicate samples, values are reported as an average and associated range. For multiplicate samples, the values are reported as an average and standard error of the mean (s.e.m.).

### Yeast **a**-factor-dependent mating assay

Qualitative and quantitative yeast mating assays were performed as detailed previously (Berger et al., 2018). In brief, SM2331, the *MAT*α strain (IH1793), and yWS164-derived *MAT***a** test strains were cultured to saturation at 25°C in YPD or selective SC− liquid media as appropriate. Cultures were normalized by dilution with fresh media (A_600_ 1.0), mixed in a 1:9 (*MAT***a**:*MAT*α) ratio in individual wells of a 96-well plate, and cell mixtures were subjected to 10-fold serial dilution using the *MAT*α cell suspension as the diluent. For qualitative analyses, each dilution series was replica spotted onto SC-lysine and minimal synthetic defined (SD) solid media. For quantitative analysis, a portion of an empirically predetermined dilution mixture was spread onto SC-lysine and SD plates in duplicate, and colonies counted after plate incubation (72 h, 30°C). The SC-lysine cell count reports the total number of *MAT***a** haploid cells initially in the mixture, whereas the SD cell count reports the number of mating events (i.e. diploids). Mating efficiencies were normalized to an **a**-factor (*MFA1*) positive control within each experiment.

### Microscopy

Imaging was performed as detailed previously (Ravishankar et al., 2023). In brief, yeast transformed with plasmids encoding GFP-RAS2-CaaX variants were cultured to late log phase (A_600_ 0.8-1) in SC-uracil liquid media and viewed using a Zeiss Axio Observer microscope equipped with fluorescence optics (Plan Apochromat 63×/1.4 N.A objective). Images were captured using AxioVision software and minor image adjustments performed using Adobe Photoshop.

### Identification and analysis of randomly sampled Ydj1-CaaX variants

yWS3186 was transformed with the Ydj1-CaaX plasmid library (pWS1775), plated onto SC-uracil solid media, and incubated for 4 days at 25°C. Randomly sampled single colonies of varying size were individually cultured and subjected to a modified temperature sensitivity assay using three spots of 20-fold serial dilutions. Growth at 41°C was scored on a scale of 0 to 5 relative to growth of the control strains yWS3312 (Ydj1-SASQ, score of 0) and yWS3311 (Ydj1-CASQ, score of 5). Plasmids were recovered from candidates scoring 0 or 5 (*n*=11 and *n*=15, respectively), sequenced to identify the CaaX sequence and retransformed into yWS3186 for retesting by temperature sensitivity (10-fold dilutions) and gel-shift assays.

### 5FOA assay

BY4741, yWS3106 and yWS3109 were transformed with combinations of *HIS3*- and *LEU2*-marked plasmids encoding *Hs*FNTA and *HsP*GGT1B, and/or empty vector plasmids, such that all strains had the same selectable markers. Strains cultured on SC-histidine-leucine-uracil solid media were used to inoculate SC-histidine-leucine liquid media. After incubation (25°C, 24 h), cultures were normalized with fresh media (A_600_ 2.0), added to wells of a 96-well plate, serially diluted with H_2_O (8-fold dilutions), and the dilution mixtures replica spotted onto SC complete solid media containing 1 mg/ml 5-FOA (Research Products International, Mount Prospect, IL, USA).

### Search for CaaX sequences

ScanProsite (https://prosite.expasy.org/scanprosite/) was used to search for CaaX sequences in *Eukaryota*, *H. sapiens*, *S. cerevisiae* and viruses in the UniProtKB/Swiss-Prot database using the search strings ‘CXXX>’, ‘C{C}XXX>’, or ‘{C}CXX>’ (search performed 12 July 2023). For compilation of Table S2, several CaaaX and CaX and sequences had no human- or virus-associated hits in the UniProtKB/Swiss-Prot database. For these sequences, the search was expanded to the UniProtKB/TrEMBL database, inclusive of all eukaryotes.

### Protein alignments

The active site residues of *Hs*FTase and rat GGTase-I have been reported previously (Reid et al., 2004). The equivalent residues for *Sc*FTase and *Sc*GGTase-I subunits were determined by structure-based sequence alignment using PyMOL and the crystal structures of *Hs*FTase (PDB: 1S63) and rat GGTase-I (PDB: 1N4Q) for reference. The following AlphaFold-derived structures were mapped onto the reference structures: *Sc*Ram1, *Sc*Ram2 and *Sc*Cdc43 (Jumper et al., 2021). EMBOSS Needle (i.e. primary sequence alignment) was used to determine the active site residues of the human GGTase-I β subunit using the rat PGGT1B sequence (Madeira et al., 2022). EMBOSS Needle was also used to determine percentage identity and percentage similarity scores for each subunit relative to the reference *Hs*FNTA, *Hs*FNTB and rat PGGT1B sequences.

## Supplementary Material

10.1242/dmm.050516_sup1 Supplementary information
