# Supplementary material for: Evaluating protein prenylation of human and viral CaaX sequences using a humanized yeast system
Source: Dis Model Mech. 2024 May 31;17(5):dmm050516. doi: 10.1242/dmm.050516 (PMC11152559; doi:10.1242/dmm.050516)
Supplement: Supplementary information [file dmm-17-050516-s1.pdf]

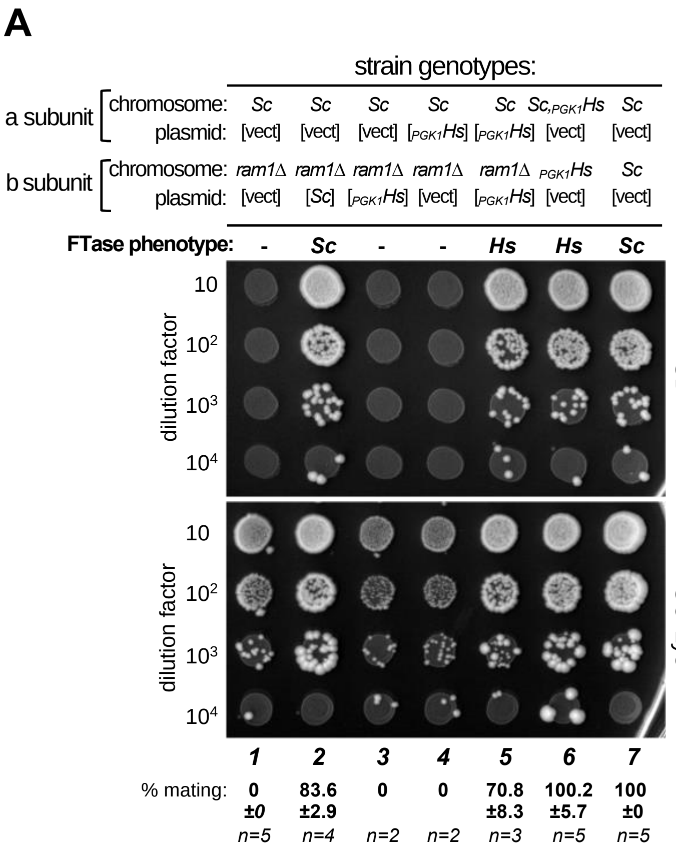


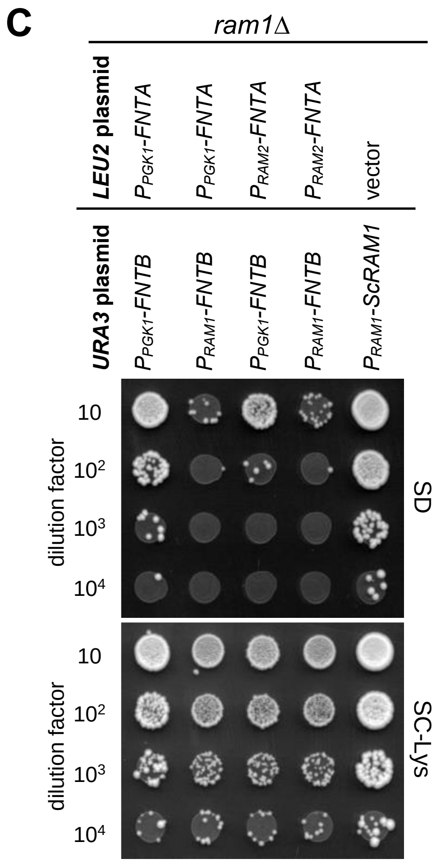


**B**

| # | relevant genotype | SD (*MATa*/a diploids) colony count | dilution plated | ml plated | colonies/ ml | SC-Lys (input *MATa* haploid) colony count | dilution plated | ml plated | colonies/ ml | mating frequency (SD/SC-Lys) | % relative to WT |
|---|---|---|---|---|---|---|---|---|---|---|---|
| 1 | *ram1Δ* | 0 | 2 | 0.2 | 0 | 660 | 10000 | 0.1 | 66000000 | 0 | 0% |
| 2 | *ram1Δ [CEN URA3 RAM1]* | 257 | 10000 | 0.3 | 8566667 | 462 | 10000 | 0.15 | 30800000 | 0.278 | 86% |
| 3 | *ram1Δ [CEN URA3 $P_{PGK1}$-FNTB]* | 0 | 2 | 0.3 | 0 | 556 | 10000 | 0.1 | 55600000 | 0 | 0% |
| 4 | *ram1Δ [CEN HIS $P_{PGK1}$-FNTA]* | 1 | 2 | 0.2 | 10 | 533 | 10000 | 0.1 | 53300000 | <0.0001 | 0% |
| 5 | *ram1Δ [CEN HIS $P_{PGK1}$-FNTA][CEN URA3 $P_{PGK1}$-FNTB]* | 108 | 10000 | 0.2 | 5400000 | 417 | 10000 | 0.15 | 27800000 | 0.194 | 60% |
| 6 | *ram1::$P_{PGK1}$-FNTB his3::HIS3-$P_{PGK1}$-FNTA* | 400 | 10000 | 0.4 | 10000000 | 391 | 10000 | 0.15 | 26066667 | 0.384 | 118% |
| 7 | *wt* | 324 | 10000 | 0.3 | 10800000 | 498 | 10000 | 0.15 | 33233333 | 0.325 | 100% |

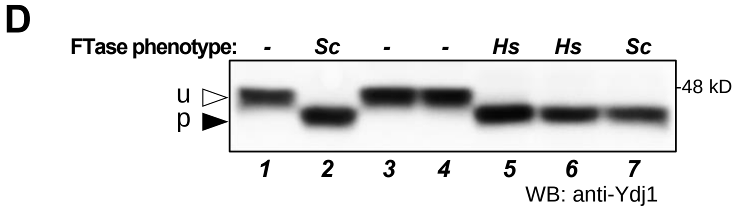


**Fig. S1. Interspecies complementation studies of FTase subunits: *Hs*FTase and *Sc*FTase subunits are not interchangeable, but co-expression of both α and β subunits of *Hs*FTase can restore FTase activity in the absence of *Sc*FTase. A)** *MAT***a** haploid strains were engineered to express yeast (*Sc*) and/or human FTase α and β subunits ($_{PGK1}$*Hs*), where subunits were encoded

at chromosomal loci (no brackets) or on plasmids (brackets). Select strains were also transformed with empty vectors (vect) so that all strains had the same selectable markers. Qualitative (SD and SC-Lys panels) and quantitative (values) mating tests were performed in parallel. For the qualitative mating assay, a representative result shown. For the quantitative mating assay, the value for the wildtype strain was set to 100% (condition ***7***), errors are standard error of the mean (SEM), and the number of biological replicates evaluated is indicated. Strains used were yWS3276 (***1***), yWS3277 (***2***), yWS3278 (***3***), yWS3408 (***4***), yWS3280 (***5***), yWS3282 (***6***), and yWS3283 (***7***). A detailed description of the assay is reported in **Fig. 2B**. **B)** Representative colony counts and calculations from a single quantitative mating test that contributed to the "% mating" data in panel **A**. See Materials and Methods and for additional assay details. **C)** *Hs*FTase subunits were encoded behind the orthologous yeast gene promoters (i.e., $P_{RAM2}$ and $P_{RAM1}$) or the constitutive phosphoglycerate kinase 1 promoter ($P_{PGK1}$) and introduced as *URA3* and *LEU2* marked plasmids into yWS3202 (*MAT***a** *ram1*Δ). The qualitative mating assay was performed as in Panel **A** and **Fig. 2B**. The experiment was performed twice. The plasmids used were pRS415, pWS1659, pWS1767, pWS1815, pWS1883, and pWS1885. See **Table S4** for plasmid details. **D**) Gel-shift analysis of Ydj1 using the strains described in panel **A**. Total cell lysates were prepared and analyzed as described in **Fig. 2D**. u – unprenylated; p – prenylated.

Disease Models & Mechanisms • Supplementary information

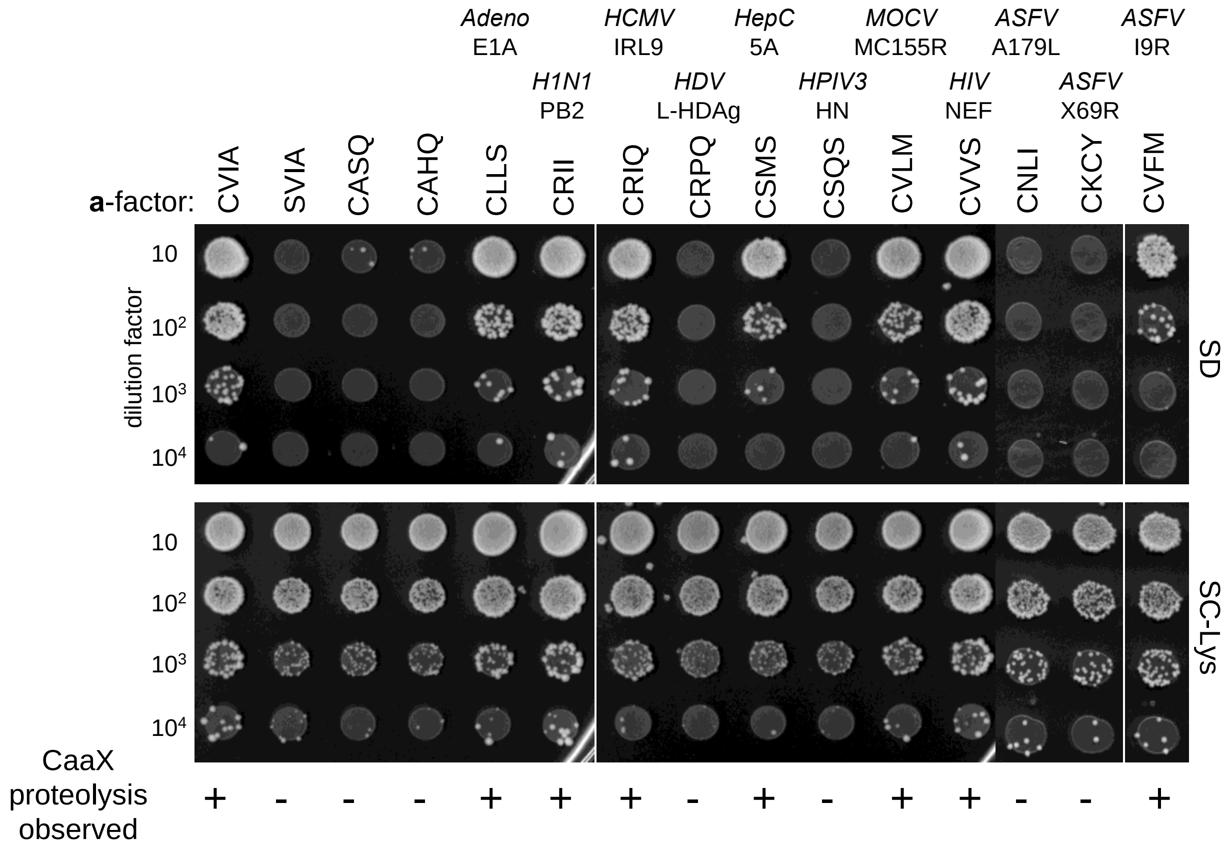


**Fig. S2. The susceptibility of viral encoded CaaX sequences to CaaX protease cleavage.** The mating assay was performed as described in **Fig. 2B** using plasmid-encoded **a**-factor-CaaX variants transformed into a yeast strain lacking chromosomal copies of the **a**-factor genes (SM2331; *MAT***a** *mfa1*Δ *mfa2*Δ). The strain expresses both yeast CaaX proteases Rce1 and Ste24, which have very similar specificities to their human counterparts (Mokry et al., 2009; Plummer et al., 2006). The viral source of the CaaX sequence is indicated above each specific sequence when applicable. Other CaaX sequences serve as controls: unprenylated (SVIA), prenylated and cleaved (CVIA), and prenylated but not cleaved (CASQ, CAHQ). Data are representative of 3 biological replicates.

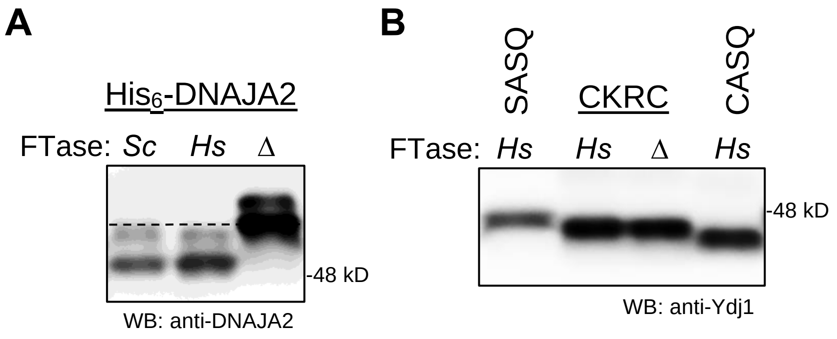


**Fig. S3**. **Gel-shift analysis of HSP40-CaaX variants with unusual gel mobilities.**
**A**) DNAJA2 exhibits major and minor bands by immunoblot. Plasmid-encoded DNAJA2 was produced in strains expressing *Sc*FTase (*Sc*; yWS2544), *Hs*FTase (*Hs*; yWS3186) or no FTase (Δ; yWS3209). Total cell lysates were prepared and analyzed as described in **Fig. 4E**. The dashed line is aligned with the main band of unprenylated DNAJA2 to better visualize that the faint band above the main band of prenylated DNAJA2 does not comigrate with unprenylated DNAJA2. **B)** Ydj1-CKRC has aberrant gel mobility and is not farnesylated by *Hs*FTase. Plasmid-encoded Ydj1-CaaX variants were produced in strains expressing *Hs*FTase (*Hs*; yWS3186) or no FTase (Δ; yWS3209). Total cell lysates were prepared and analyzed as described in **Fig. 2D**. See **Table S4** for plasmid details.

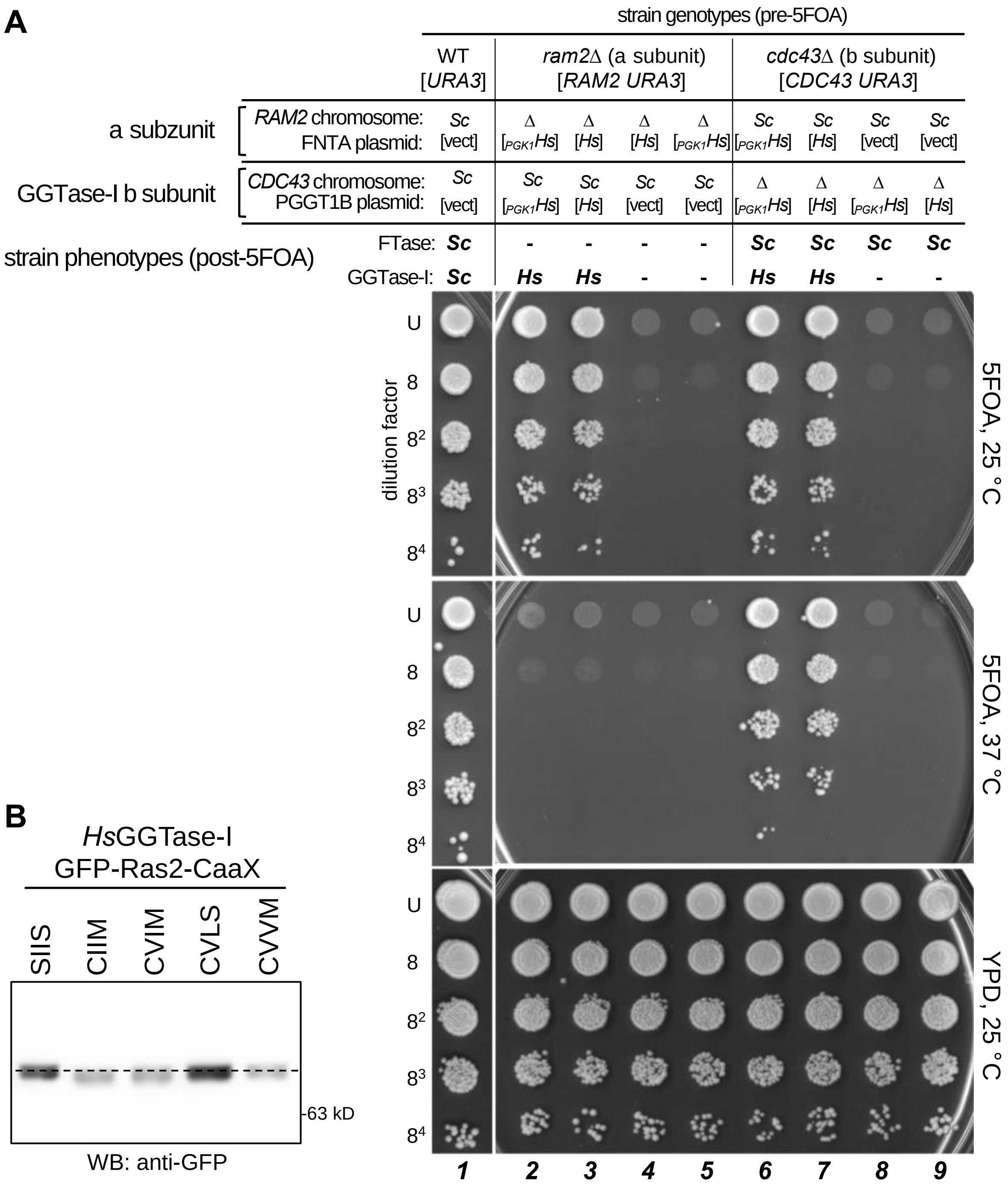


**Fig. S4. Interspecies complementation studies of GGTase-I subunits and alternate prenylation of GFP-Ras2-CVIM and CIIM.**

**A**) *Hs*GGTase-I and *Sc*GGTase-I subunits are not interchangeable, but co-expression of both α and β subunits of *Hs*GGTase-I in yeast can restore GGTase-I activity in the absence of *Sc*GGTase-I. The 5FOA assay was performed as described in **Fig. 7B**. The strains have deletions of *RAM2* (α; Δ) or *CDC43* (β; Δ), *URA3* marked plasmids encoding *RAM2* or *CDC43* to complement appropriate deletions, and various combinations of plasmids (indicated by

brackets). The plasmids encode *Hs*GGTase-I FNTA (α), PGGT1B (β), or are empty (vect). The human GGTase-I subunits are driven by either orthologous yeast gene promoters (*Hs*) or the *PGK1* promoter ($_{PGK1}$*Hs*). The plasmid combinations are such that all strains have the same selectable markers. The pre-FOA strain genotypes are indicated at the top of the panel. The post-FOA phenotypes are indicated just above the top data panel. Growth on 5FOA at 25 °C indicates the presence of functional GGTase-I (**2**, **3**, **6** and **7**). Growth on 5FOA at 37 °C depends on functional FTase, which requires α and FTase β subunits of the same species (**6** and **7**). Strains used were yWS3481 (***1***), yWS3388 (***2***), yWS3414 (***3***), yWS3639 (***4***), yWS3287 (***5***), yWS3387 (***6***), yWS3413 (***7***), yWS3285 (***8***), and yWS3638 (***9***). Data are representative of 3 biological replicates. **B**) Gel-shift analysis of GFP-Ras2-CIIM and GFP-Ras2-CVIM was performed as described in **Fig. 3A** using the humanized *Hs*GGTase-I strain yWS4215 (*ram2*Δ [*P*$_{CDC43}$-*HsPGGT1B*][ *P*$_{RAM2}$-*HsFNTA*]). Data are representative of 3 biological replicates.

Disease Models & Mechanisms • Supplementary information

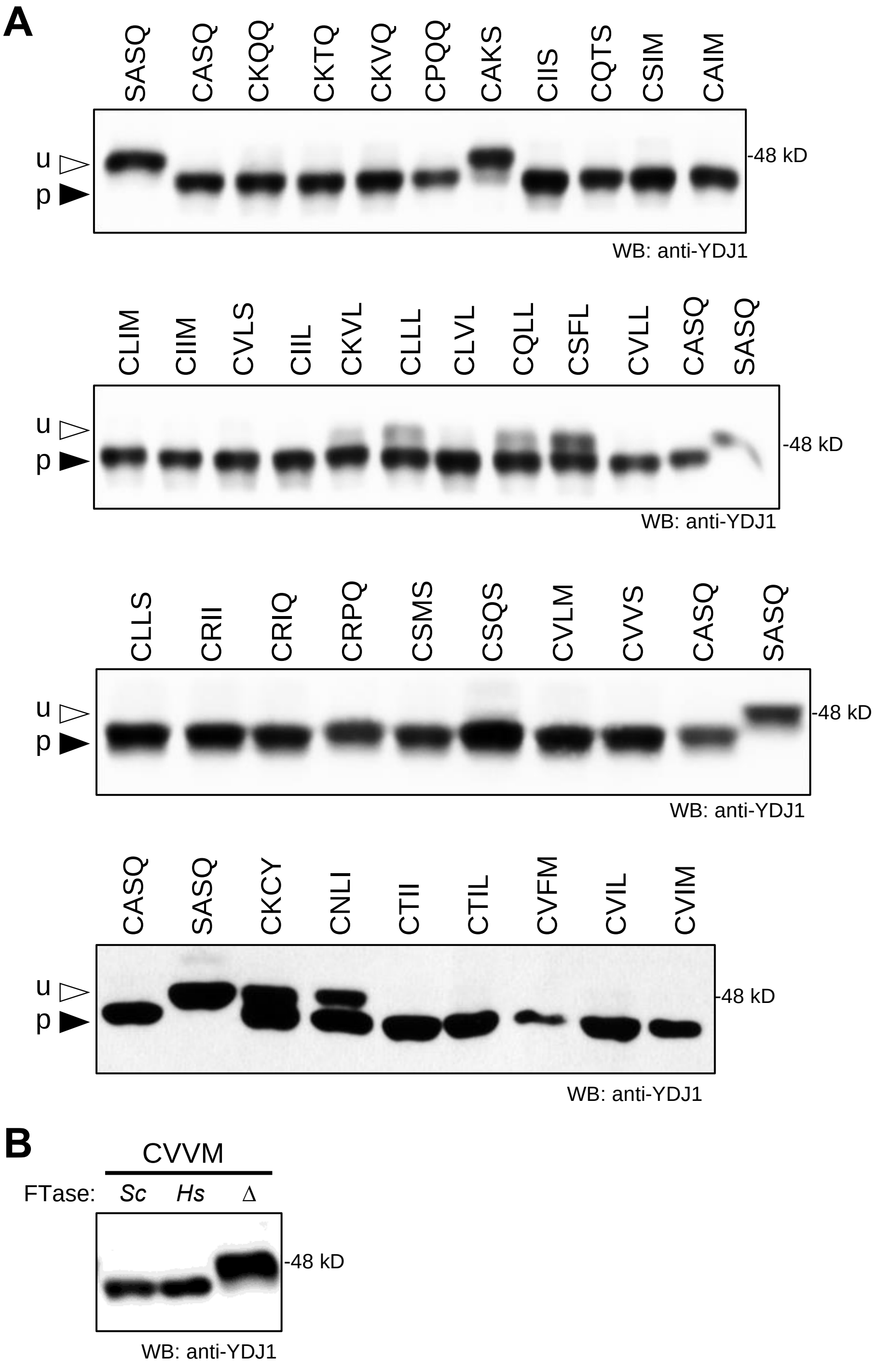


**Fig. S5. Gel-shift analysis of Ydj1-CaaX sequences described in Fig. 5 in the yeast FTase background.** Plasmid-encoded Ydj1-CaaX variants were produced in strains expressing **A**) *Sc*FTase (yWS2544) or **B**) *Sc*FTase (*Sc*; yWS2544), *Hs*FTase (*Hs*; yWS3186), or no FTase (Δ; yWS3209). Total cell lysates were prepared and analyzed as described in **Fig. 2D**. The plasmids used are listed in **Table S4**. See **Table S1** for percent prenylation calculations and number of replicates.

| | FTase/GGTase-I<br>a subunit | | FTase<br>b subunit | | GGTase-I<br>b subunit | | |
|---|---|---|---|---|---|---|---|
| Specificity | *Hs*FNTA | *Sc*Ram2 | *Hs*FNTB | *Sc*Ram1 | *Rn*PGGT1B | *Hs*PGGT1B | *Sc*Cdc43 |
| $a_2$ | - | - | Trp102<br>Trp106<br>Tyr361 | Trp108<br>Trp112<br>Tyr362 | Thr49<br>Phe53<br>Leu320 | Thr49<br>Phe53<br>Leu320 | **Ala40**<br>**Tyr44**<br>**Tyr340** |
| X | Tyr131 | Tyr66 | Leu96<br>Ala98<br>Ser99<br>Trp102<br>His149<br>Ala151<br>Pro152 | Leu102<br>Ala104<br>Ser105<br>Trp108<br>His156<br>Ala158<br>**Ser159**[a] | Thr49<br>His121<br>Ala123<br>Phe174 | Thr49<br>His121<br>Ala123<br>Phe174 | **Ala40**<br>**Asn102**<br>**Pro104**<br>Phe167 |
| % identity<br>% similarity | - | 22<br>41 | - | 34<br>48 | - | 98<br>100<br>(vs *Rn*) | 27<br>40<br>(vs *Rn*) |

[a] Subunit residues in the yeast subunit that are positionally conserved but not sequence conserved in the equivalent human subunit are bold.

**Fig. S6. Active site architectures resulting from three-dimensional alignments of human, rat, and yeast prenyltransferase subunits.**

Disease Models & Mechanisms • Supplementary information

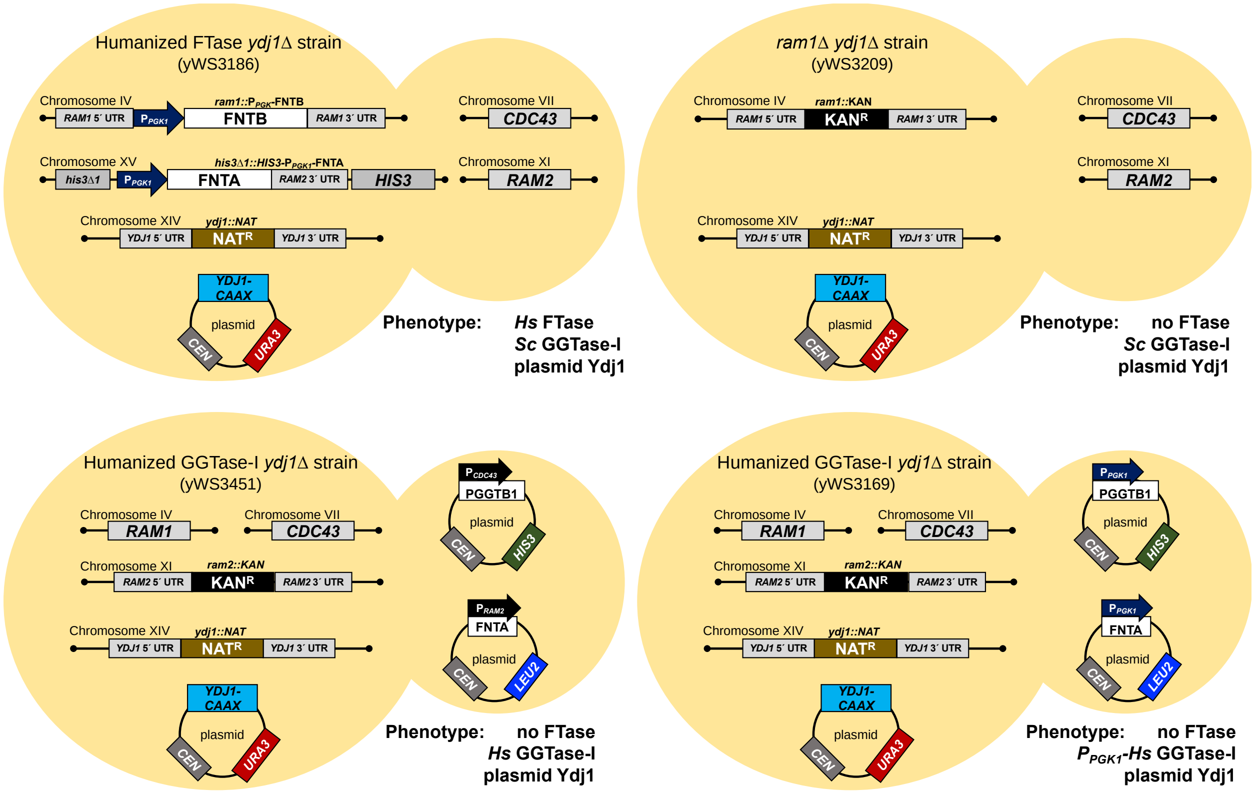


**Fig. S7. Cartoon summaries of humanized prenyltransferase strains described in this study.** The indicated strains are in addition to these reported in **Figs. 2A** and **7A**. The strain numbers, genomic architecture, associated plasmids, and relevant phenotypes are indicated on each cartoon.

**Table S1. Quantification of percent[a] prenylation by human and yeast prenyltransferases based on gel-shift analysis of indicated sequences in the context of the Ydj1 reporter.**

| | ***Hs*FTase** | ***Sc*FTase** | ***Sc*GGTase-I** | ***Hs*GGTase-I** |
|---|---|---|---|---|
| **CaaX** | **yWS3186[b]** | **yWS2544[c]** | **yWS3209[d]** | **yWS3451[e]** |
| CAHQ | 100 ± 0 (4) | 100 ± 0 (2) | 0 ± 0 (3) | 0 ± 0 (3) |
| CAIM | 100 ± 0 (3) | 100 ± 0 (2) | 33.4 ± 4.1 (2) | 37.5 ± 2.7 (3) |
| CAKS | 29.7 ± 5 (3) | 5.4 ± 3.3 (3) | 9.2 ± 9.2 (2) | ND |
| CASQ | 100 ± 0 (18) | 100 ± 0 (8) | 0 ± 0 (10) | 0 ± 0 (3) |
| CIIL | 100 ± 0 (3) | 100 ± 0 (2) | 94.4 ± 3.2 (3) | 100 ± 0 (4) |
| CIIM | 100 ± 0 (3) | 100 ± 0 (2) | 40.6 ± 3.9 (4) | 87.6 ± 2.2 (4) |
| CIIS | 100 ± 0 (3) | 100 ± 0 (2) | 6.2 ± 6.2 (2) | ND |
| CKCY | 100 ± 0 (3) | 75.1 ± 8.8 (4) | 3.6 ± 3.6 (3) | 6.4 ± 4.1 (4) |
| CKQQ | 100 ± 0 (3) | 100 ± 0 (2) | 0 ± 0 (2) | ND |
| CKTQ | 100 ± 0 (3) | 100 ± 0 (3) | 0 ± 0 (2) | ND |
| CKVL | 100 ± 0 (3) | 97.7 ± 2.3 (2) | 13.3 ± 4.4 (2) | 40.2 ± 9.2 (4) |
| CKVQ | 100 ± 0 (3) | 100 ± 0 (2) | 0 ± 0 (2) | ND |
| CLIM | 100 ± 0 (3) | 100 ± 0 (2) | 29.4 ± 6 (2) | 34.6 ± 1 (3) |
| CLLL | 95.8 ± 3 (3) | 82.9 ± 2.9 (3) | 19.4 ± 18.4 (3) | 54.9 ± 1.2 (3) |
| CLLS | 100 ± 0 (3) | 100 ± 0 (2) | 0 ± 0 (2) | ND |
| CLVL | 100 ± 0 (3) | 100 ± 0 (2) | 12.6 ± 0.6 (2) | 32.4 ± 2.7 (3) |
| CNLI | 98.3 ± 1.7 (3) | 95.3 ± 0.3 (2) | 2.7 ± 2.7 (3) | 24 ± 3.6 (4) |
| CPQQ | 100 ± 0 (3) | 100 ± 0 (2) | 0 ± 0 (2) | ND |
| CQLL | 96.5 ± 1.7 (3) | 87.1 ± 0.9 (3) | 0 ± 0 (2) | 43.2 ± 1 (3) |
| CQTS | 100 ± 0 (3) | 100 ± 0 (2) | 0 ± 0 (2) | ND |
| CRII | 100 ± 0 (4) | 100 ± 0 (2) | 17.5 ± 6.2 (3) | 75.8 ± 2.4 (3) |
| CRIQ | 100 ± 0 (4) | 100 ± 0 (2) | 1.5 ± 1.5 (3) | ND |
| CRPQ | 100 ± 0 (4) | 100 ± 0 (2) | 1.3 ± 1.3 (3) | ND |
| CSFL | 32.9 ± 1.4 (6) | 77.6 ± 5.2 (4) | 9.5 ± 2.9 (7) | 10.5 ± 0.8 (3) |
| CSIM | 100 ± 0 (3) | 100 ± 0 (2) | 23.2 ± 5.6 (2) | 30.1 ± 1.3 (3) |
| CSMS | 100 ± 0 (3) | 100 ± 0 (2) | 0 ± 0 (2) | ND |
| CSQS | 100 ± 0 (3) | 100 ± 0 (2) | 0 ± 0 (2) | ND |
| CTII | 100 ± 0 (3) | 100 ± 0 (2) | 75.2 ± 3.1 (2) | 81.4 ± 1.6 (3) |
| CTIL | 100 ± 0 (3) | 100 ± 0 (2) | 95 ± 2.6 (3) | 98.5 ± 1.5 (3) |
| CTLM | 100 ± 0 (3) | 100 ± 0 (10) | 0 ± 0 (2) | ND |
| CVFM | 100 ± 0 (3) | 100 ± 0 (3) | 3.2 ± 1.7 (4) | 9.2 ± 1.2 (3) |
| CVIA | 100 ± 0 (3) | 100 ± 0 (9) | 0 ± 0 (2) | ND |
| CVIL | 100 ± 0 (4) | 100 ± 0 (2) | 98.9 ± 1.1 (3) | 98.6 ± 1.4 (3) |
| CVIM | 99.6 ± 0.4 (6) | 100 ± 0 (3) | 41.3 ± 6 (6) | 80.1 ± 6 (4) |
| CVLL | 100 ± 0 (5) | 100 ± 0 (2) | 12.6 ± 1.5 (5) | 96 ± 2.7 (4) |
| CVLM | 100 ± 0 (3) | 100 ± 0 (2) | 1.5 ± 1.5 (2) | 58.3 ± 6.3 (3) |
| CVLS | 98.3 ± 0.6 (4) | 100 ± 0 (2) | 0 ± 0 (4) | 0 ± 0 (4) |
| CVVM | 100 ± 0 (3) | 100 ± 0 (2) | 2.4 ± 1.4 (3) | 24.1 ± 4.2 (3) |
| CVVS | 100 ± 0 (3) | 100 ± 0 (2) | 0 ± 0 (2) | ND |
| **CaX** | ***Hs*FTase** | ***Sc*FTase** | | |
| CAF | 22.7 ± 6.2 (3) | 24.1 ± 8.4 (3) | | |
| CAL | 58.9 ± 5.8 (3) | 40.2 ± 6.3 (3) | | |
| CEV | 50.2 ± 14.9 (3) | 21.2 ± 7.9 (3) | | |

Disease Models & Mechanisms • Supplementary information

| | | | | |
|---|---|---|---|---|
| CFT | 32.2 ± 8.6 (4) | 21.5 ± 7.1 (3) | | |
| CFV | 77.6 ± 7.1 (4) | 65.3 ± 4.4 (3) | | |
| CHA | 5.6 ± 2.1 (4) | 8.9 ± 0.3 (3) | | |
| CII | 80.2 ± 5.3 (3) | 74.6 ± 4.4 (3) | | |
| CLL | 14.2 ± 5.1 (3) | 33 ± 9.7 (3) | | |
| CNV | 60.8 ± 5.2 (3) | 35.9 ± 7.9 (3) | | |
| CQL | 39.8 ± 4.2 (3) | 30.1 ± 7.2 (3) | | |
| CRV | 46.5 ± 3.8 (3) | 33 ± 7.3 (3) | | |
| CSV | 68.9 ± 4.7 (3) | 26.4 ± 7.6 (3) | | |
| CTI | 88.8 ± 3.5 (3) | 53.4 ± 3.9 (3) | | |
| CTV | 68.6 ± 3.6 (3) | 44.4 ± 4.5 (3) | | |
| CVI | 95.1 ± 2.4 (4) | 78.5 ± 0.5 (3) | | |
| CVL | 27 ± 7.7 (3) | 34.2 ± 7.2 (3) | | |
| **CaaaX** | ***Hs*FTase** | ***Sc*FTase** | | |
| CASLS | 9.8 ± 4.3 (3) | 1.5 ± 1.5 (4) | | |
| CASSQ | 0 ± 0 (3) | 0 ± 0 (4) | | |
| CGGDD | 0 ± 0 (2) | 0 ± 0 (2) | | |
| CHIIM | 93.1 ± 2.6 (4) | 79.9 ± 7.1 (7) | | |
| CLACS | 5.5 ± 3.6 (3) | 0.4 ± 0.4 (4) | | |
| CLLFS | 8.1 ± 5.4 (3) | 13.8 ± 3.3 (4) | | |
| CMIGM | 20.2 ± 7.5 (3) | 39.5 ± 11.1 (5) | | |
| CMIIM | 100 ± 0 (6) | 100 ± 0 (12) | | |
| CMIIQ | 94.4 ± 3.5 (3) | 100 ± 0 (4) | | |
| CMIIS | 95.2 ± 2.4 (3) | 88.8 ± 4.9 (5) | | |
| CMKIM | 100 ± 0 (3) | 99.4 ± 0.6 (5) | | |
| CMTSQ | 5.2 ± 3.3 (3) | 0.3 ± 0.3 (4) | | |
| CQTGP | 79.9 ± 2.4 (6) | 0 ± 0 (5) | | |
| CQYNS | 0 ± 0 (3) | 0 ± 0 (4) | | |
| CSIIM | 100 ± 0 (3) | 100 ± 0 (5) | | |
| CSKLN | 0 ± 0 (3) | 11.1 ± 3.1 (4) | | |
| CSLMQ | 27.5 ± 5.6 (4) | 88.7 ± 5.9 (9) | | |
| CSQAS | 0 ± 0 (3) | 0 ± 0 (4) | | |
| CSQGP | 61 ± 2.7 (6) | 0 ± 0 (4) | | |
| CVQTS | 0 ± 0 (3) | 0.9 ± 0.9 (4) | | |
| CYIIM | 100 ± 0 (3) | 100 ± 0 (5) | | |
| **Randomly Sampled** | ***Hs*FTase** | | | |
| CCKP | 0 ± 0 (3) | | | |
| CDVY | 68.4 ± 11 (3) | | | |
| CDYQ | 100 ± 0 (3) | | | |
| CETC | 100 ± 0 (3) | | | |
| CFTP | 5.7 ± 2.4 (5) | | | |
| CGGN | 0 ± 0 (3) | | | |
| CGRT | 0 ± 0 (3) | | | |
| CHLR | 4.4 ± 4.4 (3) | | | |
| CKDD | 0 ± 0 (3) | | | |
| CKGE | 0 ± 0 (3) | | | |
| CKRC | 0 ± 0 (4) | | | |
| CNMG | 100 ± 0 (3) | | | |

Disease Models & Mechanisms • Supplementary information

| | | | | |
|---|---|---|---|---|
| CNPA | 100 ± 0 (3) | | | |
| CPAA | 98.3 ± 1.7 (3) | | | |
| CPGH | 40.4 ± 4.2 (3) | | | |
| CPMT | 100 ± 0 (3) | | | |
| CQLP | 0 ± 0 (4) | | | |
| CSNY | 42 ± 3.8 (4) | | | |
| CSTH | 76.2 ± 23.8 (3) | | | |
| CVVR | 7.1 ± 5.5 (5) | | | |
| CWFC | 91.5 ± 4.3 (3) | | | |
| CWHS | 44.3 ± 8 (3) | | | |
| CWIM | 100 ± 0 (4) | | | |
| CWNA | 32.3 ± 5.5 (4) | | | |
| CWQT | 77.7 ± 1.7 (3) | | | |
| CYME | 61.7 ± 5.5 (3) | | | |

[a] % ± error (**n** biological replicates), ND – not determined; [b]expresses *Hs*FTase and *Sc*GGTase-I (yWS3186); [c]expresses *Sc*FTase and *Sc*GGTase-I (yWS2544); [d]expresses *Sc*GGTase-I and no FTase (yWS3209); [e]expresses *Hs*GGTase-I and no FTase (yWS3451).

Disease Models & Mechanisms • Supplementary information

**Table S2. Human and viral proteins with Cxxx sequences investigated in this study.**

| CaaX | UniProtKB ID[a] | Protein Description |
|---|---|---|
| CAHQ | O60884 | DNAJA2: DnaJ homolog subfamily A member 2 |
| CAIM | P20700 | Lamin-B1 |
| CAIM | Q96S79 | RASL10B: Ras-like protein family member 10B |
| CAIM | O15498 | YKT6: Synaptobrevin homolog |
| CAIM | Q04233 (Yeast) | Gis4 |
| CAKS | P57729 | RAB38: Ras-related protein Rab-38 |
| CGGN | P04083 | ANXA1: Annexin A1 (Annexin I) |
| CIIL | Q96IG2 | FXL20: F-box/LRR-repeat protein 20 |
| CIIL | Q9UBI6 | GBG12: Guanine nucleotide-binding protein G(I)/G(S)/G(O) subunit gamma-12 |
| CIIL | O60262 | GBG7: Guanine nucleotide-binding protein G(I)/G(S)/G(O) subunit gamma-7 |
| CIIM | O95661 | DIRA3: GTP-binding protein Di-Ras3 |
| CIIM | P01116 | GTPase Kras (KRas4A) |
| CIIS | Q96MT3 | PRIC1: Prickle-like protein 1 |
| CIIS | Q7Z3G6 | PRIC2: Prickle-like protein 2 |
| CIIS | P01120 (Yeast) | Ras2 |
| CKCY | Q65134 (Swine virus) | ASFV X69R Uncharacterized membrane protein X69R |
| CKQQ | Q15831 | LKB1: Serine/threonine-protein kinase STK11 |
| CKQQ | P55209 | NAP1L1: Nucleosome assembly protein 1-like 1 |
| CKQQ | Q99733-2 | NAP1L4: Nucleosome assembly protein 1-like 4 |
| CKQQ | Q07418 (Yeast Pex19) | Pex19: Peroxisomal membrane protein import receptor Peroxin-19 |
| CKRC | O43790 | KRT86: Keratin, type II |
| CKTQ | Q02224 | CENPE: Centromere-associated protein E |
| CKTQ | Q9BVM2 | DPCD: Protein DPCD |
| CKVL | P62745 | RHOB: Rho-related GTP-binding protein |
| CKVQ | P49454 | CENPF: Centromere protein F (Mitosin) |
| CKVQ | Q9NXP7-3 | GIN1: Gypsy retrotransposon integrase-like protein 1 |
| CLIM | Q96NX5 | KCC1G: Calcium/calmodulin-dependent protein kinase type 1G |
| CLIM | P40855 | PEX19: Peroxisomal biogenesis factor 19 |
| CLLL | Q8WTQ7 | GRK7: Rhodopsin kinase |
| CLLL | P63000 | RAC1: Ras-related C3 botulinum toxin substrate 1 |
| CLLL | P62834 | RAP1A: Ras-related protein |
| CLLL | Q9HBH0 | RHOF: Rho-related GTP-binding protein |
| CLLS | P03254-3 (Human virus) | E1A: HumanAdenovirus C Early E1A protein |
| CLVL | P61586 | RHOA: Transforming protein RhoA |
| CNLI | P42485 (Swine virus) | ASFV A179L: Apoptosis regulator Bcl-2 homolog |
| CPAA | P49184 | DNSL1: Deoxyribonuclease-1-like 1 |
| CPAA | Q16363 | LAMA4: Laminin subunit alpha-4 |
| CPAA | O15230 | LAMA5: Laminin subunit alpha-5 |
| CPQQ | Q96EA4 | SPDLY: Protein Spindly |
| CQLL | P61224 | RAP1B: Ras-related protein |

Disease Models & Mechanisms • Supplementary information

| | | |
|---|---|---|
| CQTS | P31689-1 | DNAJA1: DnaJ homolog subfamily A member 1 |
| CRII | Q7T8V3 (Human virus) | Influenza Virus H1N1 Polymerase basic protein 2 |
| CRIQ | P16807 (Human virus) | Human cytomegalovirus (Human herpesvirus 5) Uncharacterized protein IRL9 |
| CRPQ | P29996 (Human virus) | L-HDAg: Hepatitis Delta Virus Large Delta Antigen |
| CSFL | P63218 | GBG5: Guanine nucleotide-binding protein G(I)/G(S)/G(O) subunit gamma-5 |
| CSIM | P02545 | LMNA: Prelamin-A/C |
| CSIM | O75781 | PALM: Paralemmin-1 |
| CSIM | Q9H0X6 | RN208: RING finger protein 208 |
| CSIM | Q92730 | RND1: Rho-related GTP-binding protein Rho6 |
| CSIM | P25378 (Yeast) | Rhb1: Rheb-related GTPase |
| CSMS | O39474 (Human virus) | Hepatitis C Virus Non-structural 5A protein |
| CSQS | O89237 (Human virus) | Human Respirovirus 3 (parainfluenza 3 virus) Hemagglutinin-neuraminidase |
| CTII | P0CAK8 (Swine virus) | ASFV protein L83L |
| CTII | P09543 | CN37: 2',3'-cyclic-nucleotide 3'-phosphodiesterase |
| CTIL | A0A0A1DY73 (Swine virus) | ASFV protein PL83L |
| CTIL | Q9UK96 | FBX10: F-box only protein 10 |
| CTIL | Q9P2W3 | GBG13: Guanine nucleotide-binding protein G(I)/G(S)/G(O) subunit gamma-13 |
| CTIL | P50150 | GBG4: Guanine nucleotide-binding protein G(I)/G(S)/G(O) subunit gamma-4 |
| CTIL | P00973-1 | OAS1: 2'-5'-oligoadenylate synthase 1 |
| CTIL | Q92834-1 | RPGR: X-linked retinitis pigmentosa GTPase regulator |
| CTLM | O95057 | DIRA1: GTP-binding protein Di-Ras1 |
| CTLM | P18852 (Yeast) | Ste18: Guanine nucleotide-binding protein subunit gamma |
| CVFM | A0A856Z113 (Swine virus) | ASFV protein I9R |
| CVIA | P34165 (Yeast) | **a**-factor (*MFA1*): Mating hormone A-factor 1 |
| CVIL | A0A0C5AZK4 (Swine virus) | ASFV Uncharacterized protein I9R |
| CVIL | Q9UKC9 | FBXL2: F-box/LRR-repeat protein 2 |
| CVIL | Q9BRT3 | MIEN1: Migration and invasion enhancer 1 |
| CVIL | Q8NBR6 | MINDY-2: Ubiquitin carboxyl-terminal hydrolase |
| CVIL | P61225 | RAP2B: Ras-related protein Rap-2b |
| CVIL | O14807 | RASM: Ras-related protein M-Ras |
| CVIL | O95164 | UBL3: Ubiquitin-like protein 3 |
| CVIM | A0A2X0TI04 (Swine virus) | ASFV uncharacterized protein I9R |
| CVIM | Q96HU8 | DIRA2: GTP-binding protein Di-Ras2 |
| CVIM | P01116-2 | RASK: GTPase Kras (KRas4B) |
| CVIM | P34226 (Yeast) | Skt5: Chitin synthase regulator |
| CVLL | A0A024RAA5 | CDC42: Cell division control protein 42 homolog |

Disease Models & Mechanisms • Supplementary information

| | | |
|---|---|---|
| CVLL | Q9UK08 | GBG8: Guanine nucleotide-binding protein G(I)/G(S)/G(O) subunit gamma-8 |
| CVLL | Q96PP8 | GBP5: Guanylate-binding protein 5 |
| CVLL | P61006 | RAB8A: Ras-related protein |
| CVLL | P10301 | RRAS: Ras-related protein |
| CVLM | Q98321 (Human virus) | MC155R: Molluscum contagiosum virus subtype 1 protein MC155R |
| CVLS | Q8N3D4 | EH1L1: EH domain-binding protein 1-like protein 1 |
| CVLS | P01112-1 | RASH: GTPase HRas |
| CVVM | Q8IXS6 | PALM2: Paralemmin-2 |
| CVVM | P01111 | GTPase NRas/ Transforming protein N-Ras |
| CVVS | Q9QRB0 (Human virus) | HIV NEF Protein |
| CVVS | A6NHY2 | AKD1B: Ankyrin repeat and death domain-containing protein 1B |
| CVVS | A0A0B4J240 | TVA10: T cell receptor alpha variable 10 |
| CVVS | A0A0B4J237 | TVA82: T cell receptor alpha variable 8-2 |
| | | |
| **CaX** | | |
| CAF | P36551-2 | COX: Isoform 2 of Oxygen-dependent coproporphyrinogen-III oxidase, mitochondrial |
| CAF | Q8NI17-11 | IL-31RA: Interleukin-31 receptor subunit alpha |
| CAF | A0A0B4J272 | TVA24: T cell receptor alpha variable 24 |
| CAF | A0JD37 | T cell receptor delta variable 3 |
| CAL | Q9UKA8 | RCAN3: Calcipressin-3 |
| CAL | Q9UKA8-2 | RCAN3: Calcipressin-3 |
| CAL | Q9UKA8-5 | RCAN3: Calcipressin-3 |
| CAL | Q9UKA8-6 | RCAN3: Calcipressin-3 |
| CAL | Q1HG43 | DOXA1: Dual oxidase maturation factor 1in-3 |
| CAL | Q6IA86 | ELP2: Elongator complex protein 2 |
| CAL | Q6IA86-2 | ELP2: Elongator complex protein 2 |
| CAL | Q6IA86-3 | ELP2: Elongator complex protein 2 |
| CAL | Q6IA86-7 | ELP2: Elongator complex protein 2 |
| CAL | Q6IA86-6 | ELP2: Elongator complex protein 2 |
| CAL | Q6IA86-5 | ELP2: Elongator complex protein 2 |
| CAL | O15504-2 | NUP42: Nucleoporin |
| CAL | Q9NUI1-2 | DECR2: Peroxisomal 2,4-dienoyl-CoA reductase [(3E)-enoyl-CoA-producing] |
| CAL | Q1HG43-3 | DOXA1: Dual oxidase maturation factor 1 |
| CAL | P08543 (Human virus) | RIR1: Ribonucleoside-diphosphate reductase large subunit-Human herpesvirus 1 |
| CAL | P09248 (Human virus) | RIR1: Ribonucleoside-diphosphate reductase large subunit-Human Varicella-zoster virus |
| CAL | P09853 (Human virus) | RIR1: Ribonucleoside-diphosphate reductase large subunit-Human herpesvirus 2 |
| CAL | P89462 (Human virus) | RIR1: Ribonucleoside-diphosphate reductase large subunit-Human herpesvirus 2 |
| CAL | Q4JQV6 (Human virus) | RIR1: Ribonucleoside-diphosphate reductase large subunit-Human Varicella-zoster viru |
| CEV | P56559-2 | ARL4C: ADP-ribosylation factor-like protein 4C |

| | | |
|---|---|---|
| CEV | Q7L8W6-2 | DPH6: Diphthine--ammonia ligase |
| CEV | Q86UE6 | LRRT1: Leucine-rich repeat transmembrane neuronal protein 1 |
| CEV | O43300 | LRRT2: Leucine-rich repeat transmembrane neuronal protein 2 |
| CEV | Q86VH5-2 | LRRT3: Leucine-rich repeat transmembrane neuronal protein 3 |
| CEV | Q86VH4-2 | LRRT4: Leucine-rich repeat transmembrane neuronal protein 4 |
| CFT | Q8N957-1 | ANKF1: Ankyrin repeat and fibronectin type-III domain-containing protein 1 |
| CFT | Q13111-2 | CAF1A: Isoform 2 of Chromatin assembly factor 1 subunit A |
| CFT | O15440-2 | MRP5: ATP-binding cassette sub-family C member 5 |
| CFV | P05997 | CO5A2: Collagen alpha-2(V) chain |
| CFV | Q96L33 | RHOV: Rho-related GTP-binding protein RhoV |
| CFV | Q7L0Q8-1 | RHOU: Rho-related GTP-binding protein RhoU |
| CHA | P0DTD8 (Human virus) | NS7B: Sars-Cov2 orf7b protein |
| CII | Q5T1H1-2 | EYS: Protein eyes shut homolog |
| CII | Q96CV9 | OPTN: Optineurin |
| CII | Q96CV9-2 | OPTN: Optineurin |
| CII | Q96CV9-3 | OPTN: Optineurin |
| CII | Q6WBX8 | RAD9B: Cell cycle checkpoint control protein |
| CII | Q6WBX8-4 | RAD9B: Cell cycle checkpoint control protein |
| CII | P82675-2 | RT05: 28S ribosomal protein S5, mitochondrial |
| CLL | Q6ZUX7 | LHFPL: tetraspan subfamily member 2 protein |
| CLL | Q9NVC6 | MED17: Mediator of RNA polymerase II transcription subunit 17 |
| CLL | Q75NE6 | MIRH1: Putative microRNA 17 host gene protein |
| CLL | Q00765-2 | REEP5: Receptor expression-enhancing protein 5 |
| CLL | Q7Z2W9 | RM21: 39S ribosomal protein L21, mitochondrial |
| CLL | Q7Z2W9-2 | RM21: 39S ribosomal protein L21, mitochondrial |
| CLL | Q96I59 | SYNM: Probable asparagine--tRNA ligase, mitochondrial |
| CLL | Q96I59-2 | SYNM: Probable asparagine--tRNA ligase, mitochondrial |
| CNV | Q9NJP7 (Scorpion toxin) | KAX91: Potassium channel toxin alpha-KTx 9.1 |
| CQL | Q8WXX0-4 | DYH7: Dynein axonemal heavy chain 7 |
| CQL | P55082 | MFAP3: Microfibril-associated glycoprotein 3 |
| CQL | P55082-2 | MFAP3: Microfibril-associated glycoprotein 3 |
| CQL | Q8N114-4 | SHSA5: Protein shisa-5 |
| CRV | Q9Y2C4-2 | EXOG: Nuclease EXOG, mitochondrial |
| CRV | F5HB62 (Human virus) | KITH: Thymidine kinase-Human herpes virus |
| CSV | Q9UEW3 | MARCO: Macrophage receptor |
| CSV | Q9UEW3-2 | MARCO: Macrophage receptor |
| CSV | Q9H3H1 | MOD5: tRNA dimethylallyltransferase |
| CSV | Q9H3H1-2 | MOD5: tRNA dimethylallyltransferase |
| CSV | Q9H3H1-3 | MOD5: tRNA dimethylallyltransferase |
| CSV | Q9H3H1-4 | MOD5: tRNA dimethylallyltransferase |
| CSV | Q9H3H1-5 | MOD5: tRNA dimethylallyltransferase |
| CSV | Q9H3H1-6 | MOD5: tRNA dimethylallyltransferase |
| CSV | A8MW92 | P20L1: PHD finger protein 20-like protein 1 |
| CSV | Q9UKA9-5 | PTBP2: Polypyrimidine tract-binding protein 2 |
| CSV | Q9UKA9-6 | PTBP2: Polypyrimidine tract-binding protein 2 |

Disease Models & Mechanisms • Supplementary information

| | | |
|---|---|---|
| CSV | O95969 | SG1D2: Secretoglobin family 1D member 2 (Lipophilin-B) |
| CTI | P08998 (Chicken) | SOMA: Somatotropin growth hormone |
| CTV | A6H8Y1-2 | BDP1: Transcription factor TFIIIB component B" homolog |
| CTV | O75044 | SRGP2: SLIT-ROBO Rho GTPase-activating protein 2 |
| CVI | F5H8R0 (Human virus) | U147A: Human Cytomegalovirus protein UL147A |
| CVI | P60893 | GPR85: Probable G-protein coupled receptor 85 |
| CVL | Q32MH5-2 | ATOSA: Atos homolog protein A |
| CVL | P23510 | TNFL4: Tumor necrosis factor ligand superfamily member 4 |
| CVL | P23510-2 | TNFL4: Tumor necrosis factor ligand superfamily member 4 |
| | | |
| **CaaaX** | | |
| CASLS | Q6MZQ0 | PRR5L: Proline-rich protein 5-like |
| CASSQ | A0A5B0 | TVB14: T cell receptor beta variable 14 |
| CASSQ | A0A087WV62 | TVB16: T cell receptor beta variable 16 |
| CASSQ | A0A0A0MS06 | TVB23: Probable non-functional T cell receptor beta variable 23-1 |
| CASSQ | A0A576 | TVB31: T cell receptor beta variable 3-1 |
| CASSQ | A0A577 | TVB41: T cell receptor beta variable 4-1 |
| CASSQ | A0A539 | TVB42: T cell receptor beta variable 4-2 |
| CASSQ | A0A589 | TVB43: T cell receptor beta variable 4-3 |
| CHIIM | NO HITS | |
| CLACS | O75094-1 | SLIT3: Slit homolog 3 protein |
| CLLFS | Q14980-3 | NUMA1: Nuclear mitotic apparatus protein 1 |
| CMIGM | NO HITS | |
| CMIIM | NO HITS | |
| CMIIQ | NO HITS | |
| CMIIS | A0A8C5HAK0 (Clingfish) | Transcriptional adapter 1 |
| CMKIM | NO HITS | |
| CMTSQ | Q00973 | B4GN1: Beta-1,4 N-acetylgalactosaminyltransferase 1 |
| CQTGP | Q8IVK1-1 | GLCM1: Putative glycosylation-dependent cell adhesion molecule 1; GlyCAM-1 |
| CQYNS | P78337 | PITX1: Pituitary homeobox 1 |
| CSIIM | A0A183M795 (Flatworm) | MANSC domain-containing protein |
| CSKLN | O60285-1 | NUAK1: NUAK family SNF1-like kinase 1 |
| CSKLN | P0C604 | OR4A8: Olfactory receptor 4A8 |
| CSLMQ | O75764-2 | TCEA3: Transcription elongation factor A protein 3 |
| CSQAS | Q9NQH7 | XPP3: Xaa-Pro aminopeptidase 3 |
| CSQGP | Q8TER0-5 | SNED1: Sushi, nidogen and EGF-like domain-containing protein 1 |
| CVQTS | P0CG34 | TB15A: Thymosin beta-15A |
| CVQTS | P0CG35 | TB15B: Thymosin beta-15B |
| CYIIM | NO HITS | |

[a]Human protein unless otherwise noted.

**Table S3. Yeast strains used in this study.**

| Strain ID | Genotype | Reference | Plasmids |
|---|---|---|---|
| BY4741 | *MAT***a** *his3Δ1 leu2Δ0 met15Δ0 ura3Δ0* | (Brachmann et al., 1998) | |
| BY4742 | *MATα his3Δ1 leu2Δ0 lys2Δ0 ura3Δ0* | this study | |
| BY4743 | *MAT***a***/α his3Δ1 leu2Δ0 met15Δ0 ura3Δ0/his3Δ1 leu2Δ0 lys2Δ0 ura3Δ0; cdc43::KAN$^R$/CDC43* | Dharmacon CloneId:24522 | |
| BY4743 | *MAT***a***/α his3Δ1 leu2Δ0 met15Δ0 ura3Δ0/his3Δ1 leu2Δ0 lys2Δ0 ura3Δ0; ram2::KAN$^R$/RAM2* | Dharmacon CloneId:24868 | |
| SM1068 | *MATα lys1* | (Cadinanos et al., 2003) | |
| SM2331 | *MAT***a** *trp1 leu2 ura3 his4 can1 mfa1-Δ1 mfa2-Δ1* | *(Chen et al., 1997)* | |
| yWS1632 | *MAT***a** *his3Δ1 leu2Δ0 met15Δ0 ura3Δ0 ram1::KAN$^R$ ρ–* | (Shoemaker et al., 1996) | |
| yWS164 | *MAT***a** *trp1 leu2 ura3 his4 can1 mfa1-Δ1 mfa2-Δ1 rce1::TRP1 ste24::KAN$^R$* | (Cadinanos et al., 2003) | |
| yWS2542 | *MAT***a** *his3Δ1 leu2Δ0 met15Δ0 ura3Δ0 ram1::KAN ydj1::NAT$^R$* | (Berger et al., 2018) | |
| yWS2544 | *MAT***a** *his3Δ1 leu2Δ0 met15Δ0 ura3Δ0 ydj1::NAT$^R$* | (Berger et al., 2018) | |
| yWS3104 | *MAT***a** *his3Δ1 leu2Δ0 met15Δ0 ura3Δ0 ram1::P$_{PGK1}$-FNTB ydj1::NAT$^R$* | this study | |
| yWS3105 | *MAT***a** *his3Δ1 leu2Δ met15Δ ura3Δ ram1::P$_{PGK1}$-FNTB* | this study | |
| yWS3106 | *MAT***a** *his3Δ1 leu2Δ0 met15Δ0 ura3Δ0 cdc43::KAN$^R$ [CEN URA3 CDC43]* | this study | pWS1651 |
| yWS3109 | *MAT***a** *his3Δ1 leu2Δ0 met15Δ0 ura3Δ0 ram2::KAN$^R$ [CEN URA3 RAM2]* | this study | pWS1277 |
| yWS3131 | *MAT***a** *his3Δ1 leu2Δ0 met15Δ0 ura3Δ0 ram2::KAN$^R$ [CEN HIS3 P$_{PGK1}$-FNTA][CEN LEU2 P$_{PGK1}$-PGGT1B]* | this study | pWS1903, pWS1934 |
| yWS3132 | *MAT***a** *leu2Δ0 met15Δ0 ura3Δ0 ram1::P$_{PGK1}$-FNTB his3Δ1::HIS3-P$_{PGK1}$-FNTA ρ–* | this study | |
| yWS3134 | *MAT***a** *leu2Δ0 met15Δ0 ura3Δ0 ram1:: P$_{PGK1}$-FNTB ydj1::NAT$^R$ his3Δ1::HIS3- P$_{PGK1}$-FNTA ρ–* | this study | |
| yWS3169 | *MAT***a** *his3Δ1 leu2Δ0 met15Δ0 ura3Δ0 ram2::KAN ydj1::NAT$^R$ [CEN HIS3 P$_{PGK1}$-FNTA][CEN LEU2 P$_{PGK1}$-PGGT1B]* | this study | pWS1903, pWS1934 |
| yWS3186 | *MAT***a** *leu2Δ0 met15Δ0 ura3Δ0 ram1::P$_{PGK1}$-FNTB ydj1::NAT$^R$ his3Δ1::HIS3-P$_{PGK1}$-FNTA* | this study | |
| yWS3202 | *MAT***a** *his3Δ1 leu2Δ0 met15Δ0 ura3Δ0 ram1::KAN$^R$* | this study | |
| yWS3209 | *MAT***a** *his3Δ1 leu2Δ0 ura3Δ0 ram1::KAN$^R$ ydj1::NAT$^R$* | this study | |
| yWS3220 | *MAT***a** *leu2Δ0 met15Δ0 ura3Δ0 ram1::P$_{PGK1}$-FNTB his3Δ1::HIS3-P$_{PGK1}$-FNTA* | this study | |
| yWS3276 | yWS3202 *[CEN HIS3][CEN URA3]* | this study | pRS413, pRS416 |
| yWS3277 | yWS3202 *[CEN HIS3][CEN URA3 RAM1]* | this study | pRS413, pWS1767 |

Disease Models & Mechanisms • Supplementary information

| | | | |
|---|---|---|---|
| yWS3278 | yWS3202 *[CEN HIS3][CEN URA3 $P_{PGK1}$-HsFNTB]* | this study | pRS413, pWS1883 |
| yWS3280 | yWS3202 *[CEN HIS3 $P_{PGK1}$-HsFNTA][CEN URA3 $P_{PGK1}$-HsFNTB]* | this study | pWS1902, pWS1883 |
| yWS3282 | yWS3220 *[CEN HIS3][CEN URA3]* | this study | pRS413, pRS416 |
| yWS3283 | BY4741 *[CEN HIS3][CEN URA3]* | this study | pRS413, pRS416 |
| yWS3285 | yWS3106 *[CEN HIS3][CEN LEU2 $P_{PGK1}$-HsPGGT1B]* | this study | pWS1651, pWS1934, pRS413 |
| yWS3287 | yWS3109 *[CEN LEU2][CEN HIS3 $P_{PGK1}$-FNTA]* | this study | pWS1277, pRS415, pWS1903 |
| yWS3387 | yWS3106 *[CEN HIS3 $P_{PGK1}$-FNTA][CEN LEU2 $P_{PGK1}$-PGGT1B]* | this study | pWS1651, pWS1934, pWS1903 |
| yWS3388 | yWS3109 *[CEN HIS3 $P_{PGK1}$-FNTA][CEN LEU2 $P_{PGK1}$-PGGT1B]* | this study | pWS1277, pWS1934, pWS1903 |
| yWS3408 | yWS3202 *[CEN URA3][CEN HIS3 $P_{PGK1}$-HsFNTA]* | this study | pRS416, pWS1902 |
| yWS3411 | yWS3109 [CEN HIS3][CEN LEU2] | this study | pWS1277 pRS413 pRS415 |
| yWS3413 | yWS3106 *[CEN HIS3 $P_{CDC43}$-HsPGGT1B][CEN LEU2 $P_{RAM2}$-HsFNTA]* | this study | pWS1651, pWS1815, pWS2058 |
| yWS3414 | yWS3109 *[CEN HIS3 $P_{CDC43}$-HsPGGT1B][CEN LEU2 $P_{RAM2}$-HsFNTA]* | this study | pWS1277, pWS1815, pWS2058 |
| yWS3451 | *MAT***a** *his3Δ1 leu2Δ0 met15Δ0 ura3Δ0 ram2::KAN ydj1::NAT$^R$ [CEN LEU2 $P_{RAM2}$-HsFNTA][CEN HIS3 $P_{CDC43}$-HsPGGT1B]* | this study | pWS1815, pWS2058 |
| yWS3481 | BY4741 *[CEN HIS3][CEN LEU2][CEN URA3]* | this study | pRS416, pRS415, pRS413 |
| yWS3638 | yWS3106 *[CEN LEU2][CEN HIS3 $P_{CDC43}$-HsPGGT1B]* | this study | pWS1651, pRS415, pWS2058 |
| yWS3639 | yWS3109 *[CEN LEU2 $P_{RAM2}$-HsFNTA][CEN HIS3]* | this study | pWS1277, pWS1815, pRS413 |
| yWS4215 | *MAT***a** *his3Δ1 leu2Δ0 met15Δ0 ura3Δ0 ram2::KAN$^R$ [CEN HIS3 $P_{CDC43}$-HsPGGT1B][CEN LEU2 $P_{RAM2}$-HsFNTA]* | this study | pWS1815, pWS2058 |

**Table S4. Plasmids used in this study.**

| **Plasmid Name** | **plasmid description** | **Reference** |
|---|---|---|
| #19546 | Hygromycin $His_6$-DNAJA2 | Addgene Plasmid #19546 |
| p-05547 | *2µ LEU2 Myc-HRasQ61L* | (Stein et al., 2015) |
| pRS316 | *CEN URA3* | (Sikorski and Hieter, 1989) |
| pRS413 | *CEN HIS3* | (Sikorski and Hieter, 1989) |
| pRS415 | *CEN LEU2* | (Sikorski and Hieter, 1989) |
| pRS416 | *CEN URA3* | (Sikorski and Hieter, 1989) |
| pWS335 | *2µ URA3* $P_{PGK1}$-$His_{10}$-*HA-Hs Rce1Δ22* | (Plummer et al., 2006) |
| pWS610 | *CEN LEU2 MFA1* | (Krishnankutty et al., 2009) |
| pWS613 | *CEN LEU2 MFA1-CTLM* | (Hildebrandt et al., 2016) |
| pWS727 | *CEN LEU2 MFA1-CKQQ* | (Krishnankutty et al., 2009) |
| pWS942 | *CEN URA3 YDJ1* | (Hildebrandt et al., 2016) |
| pWS948 | *2µ URA3* $P_{PGK1}$-*YDJ1* | (Hildebrandt et al., 2016) |
| pWS965 | *CEN URA3* $P_{PGK1}$-$His_{10}$-*HA-HsRce1Δ22* | this study |
| pWS1132 | *CEN URA3 YDJ1-SASQ* | (Hildebrandt et al., 2016) |
| pWS1171 | *CEN URA3 YDJ1-CTI* | (Ashok et al., 2020) |
| pWS1172 | *CEN URA3 YDJ1-CII* | (Ashok et al., 2020) |
| pWS1173 | *CEN URA3 YDJ1-CFV* | (Ashok et al., 2020) |
| pWS1174 | *CEN URA3 YDJ1-CVI* | (Ashok et al., 2020) |
| pWS1181 | *CEN URA3 YDJ1-CNV* | (Ashok et al., 2020) |
| pWS1209 | *CEN URA3 YDJ1-CAL* | (Ashok et al., 2020) |
| pWS1210 | *CEN URA3 YDJ1-CLL* | (Ashok et al., 2020) |
| pWS1211 | *CEN URA3 YDJ1-CSV* | (Ashok et al., 2020) |
| pWS1212 | *CEN URA3 YDJ1-CQL* | (Ashok et al., 2020) |
| pWS1213 | *CEN URA3 YDJ1-CAF* | (Ashok et al., 2020) |
| pWS1214 | *CEN URA3 YDJ1-CVL* | (Ashok et al., 2020) |
| pWS1216 | *CEN URA3 YDJ1-CEV* | (Ashok et al., 2020) |
| pWS1218 | *CEN URA3 YDJ1-CFT* | (Ashok et al., 2020) |
| pWS1221 | *CEN URA3 YDJ1-CTV* | (Ashok et al., 2020) |
| pWS1246 | *CEN URA3 YDJ1-CTLM* | (Hildebrandt et al., 2016) |
| pWS1277 | *CEN URA3 RAM2* | this study |
| pWS1278 | *CEN URA3 RAM2* | this study |
| pWS1286 | *CEN URA3 YDJ1-CVIA* | (Hildebrandt et al., 2016) |
| pWS1321 | *CEN URA3 YDJ1-CVLL* | this study |
| pWS1365 | *CEN URA3 YDJ1-CRV* | this study |
| pWS1407 | *CEN URA3 YDJ1-CKQQ* | this study |
| pWS1408 | *CEN URA3 YDJ1-CGGDD* | (Blanden et al., 2018) |
| pWS1424 | *CEN URA3* $P_{YDJ1}$-$His_6$-*DNAJA2* | this study |
| pWS1437 | *CEN URA3 YDJ1-CAHQ* | (Berger et al., 2022) |
| pWS1454 | *CEN URA3 YDJ1-CAIM* | this study |
| pWS1455 | *CEN URA3 YDJ1-CAKS* | this study |
| pWS1456 | *CEN URA3 YDJ1-CIIS* | (Berger et al., 2022) |
| pWS1457 | *CEN URA3 YDJ1-CKTQ* | this study |
| pWS1458 | *CEN URA3 YDJ1-CKVQ* | this study |
| pWS1459 | *CEN URA3 YDJ1-CLIM* | this study |
| pWS1460 | *CEN URA3 YDJ1-CQTS* | (Berger et al., 2022) |
| pWS1461 | *CEN URA3 YDJ1-CSFL* | (Berger et al., 2022) |

| | | |
|---|---|---|
| pWS1462 | *CEN URA3 YDJ1-CSIM* | this study |
| pWS1463 | *CEN URA3 YDJ1-CVIM* | (Berger et al., 2022) |
| pWS1464 | *CEN URA3 YDJ1-CVLS* | this study |
| pWS1466 | *CEN URA3 YDJ1-CPQQ* | this study |
| pWS1471 | *CEN URA3 YDJ1-CQTGP* | (Blanden et al., 2018) |
| pWS1472 | *CEN URA3 YDJ1-CSQGP* | this study |
| pWS1488 | *CEN URA3 YDJ1-CMIIM* | (Blanden et al., 2018) |
| pWS1561 | *CEN LEU2 MFA1-CSIM* | (Berger et al., 2022) |
| pWS1609 | *CEN URA3 $P_{PGK1}$-HsSTE24* | (Berger et al., 2022) |
| pWS1635 | *CEN URA3 YDJ1-CVIL* | this study |
| pWS1651 | *CEN URA3 CDC43* | this study |
| pWS1655 | *Hs*FNTA in pBluescript II KS(-) | GenScript |
| pWS1656 | *Hs*FNTB in pBluescript II KS(-) | GenScript |
| pWS1657 | *Hs*PGGT1B in pBluescript II KS(-) | GenScript |
| pWS1658 | *CEN URA3 $P_{RAM2}$-HsFNTA* | this study |
| pWS1659 | *CEN URA3 $P_{RAM1}$-HsFNTB* | this study |
| pWS1660 | *CEN URA3 HsGGT1B* | this study |
| pWS1661 | *CEN LEU2 MFA1-CLIM* | this study |
| pWS1662 | *CEN LEU2 MFA1-CAIM* | this study |
| pWS1719 | *CEN LEU2 $P_{RAM1}$-HsFNTB* | this study |
| pWS1728 | *CEN URA3 YDJ1-SASQ-BsrGI* | this study |
| pWS1735 | *CEN URA3 $P_{YDJ1}$-GFP-RAS2-CCIIS* | (Ravishankar et al., 2023) |
| pWS1751 | *CEN URA3 YDJ1-CIIL* | (Berger et al., 2022) |
| pWS1767 | *CEN URA3 RAM1* | this study |
| pWS1775 | *CEN URA3 YDJ1-Cxxx* | (Kim et al., 2023) |
| pWS1814 | *CEN URA3 YDJ1-SVI* | (Ashok et al., 2020) |
| pWS1815 | *CEN LEU2 $P_{RAM2}$-HsFNTA* | this study |
| pWS1829 | *CEN URA3 YDJ1-CTIL* | this study |
| pWS1830 | *CEN URA3 YDJ1-CNLI* | (Berger et al., 2022) |
| pWS1831 | *CEN URA3 YDJ1-CKCY* | this study |
| pWS1833 | *CEN URA3 YDJ1-CTII* | this study |
| pWS1834 | *CEN URA3 YDJ1-CVFM* | (Berger et al., 2022) |
| pWS1860 | *CEN URA3 YDJ1-CHA* | this study |
| pWS1861 | *CEN URA3 $P_{PGK1}$-HsFNTA* | this study |
| pWS1862 | *CEN URA3 $P_{PGK1}$-HsFNTA* | this study |
| pWS1863 | *CEN LEU2 $P_{PGK1}$-HsFNTB* | this study |
| pWS1874 | *CEN URA3 YDJ1-SHA* | this study |
| pWS1883 | *CEN URA3 $P_{PGK1}$-HsFNTB* | this study |
| pWS1885 | *CEN LEU2 $P_{PGK1}$-HsFNTA* | this study |
| pWS1889 | *CEN URA3 $P_{YDJ1}$-GFP-RAS2-SSIIS* | (Ravishankar et al., 2023) |
| pWS1902 | *CEN HIS3 $P_{PGK1}$-HsFNTA* | this study |
| pWS1903 | *CEN HIS3 $P_{PGK1}$-HsFNTA* | this study |
| pWS1914 | *CEN URA3 $P_{PGK1}$-HsPGGT1B* | this study |
| pWS1917 | *CEN URA3 YDJ1-SMIIM* | (Schey et al., 2021) |
| pWS1918 | *CEN URA3 YDJ1-CMIIS* | (Schey et al., 2021) |
| pWS1919 | *CEN URA3 YDJ1-CMIIQ* | (Schey et al., 2021) |
| pWS1920 | *CEN URA3 YDJ1-CHIIM* | (Schey et al., 2021) |
| pWS1921 | *CEN URA3 YDJ1-CYIIM* | (Schey et al., 2021) |
| pWS1922 | *CEN URA3 YDJ1-CSIIM* | (Schey et al., 2021) |

| | | |
|---|---|---|
| pWS1923 | *CEN URA3 YDJ1-CMKIM* | (Schey et al., 2021) |
| pWS1924 | *CEN URA3 YDJ1-CMIGM* | (Schey et al., 2021) |
| pWS1934 | *CEN LEU2* $P_{PGK1}$*-HsPGGT1B* | this study |
| pWS1981 | *CEN URA3 YDJ1-CSLMQ* | (Schey et al., 2021) |
| pWS1982 | *CEN URA3 YDJ1-CSQAS* | (Schey et al., 2021) |
| pWS1983 | *CEN URA3 YDJ1-CLLFS* | (Schey et al., 2021) |
| pWS1984 | *CEN URA3 YDJ1-CVQTS* | (Schey et al., 2021) |
| pWS1985 | *CEN URA3 YDJ1-CQYNS* | (Schey et al., 2021) |
| pWS1986 | *CEN URA3 YDJ1-CLACS* | (Schey et al., 2021) |
| pWS1987 | *CEN URA3 YDJ1-CMTSQ* | (Schey et al., 2021) |
| pWS1988 | *CEN URA3 YDJ1-CASLS* | (Schey et al., 2021) |
| pWS1989 | *CEN URA3 YDJ1-CASSQ* | (Schey et al., 2021) |
| pWS1990 | *CEN URA3 YDJ1-CSKLN* | (Schey et al., 2021) |
| pWS1997 | *CEN URA3* $P_{YDJ1}$*-GFP-RAS2-CIIM* | this study |
| pWS1998 | *CEN URA3* $P_{YDJ1}$*-GFP-RAS2-CVVM* | this study |
| pWS1999 | *CEN URA3* $P_{YDJ1}$*-GFP-RAS2-CVLS* | this study |
| pWS2000 | *CEN URA3* $P_{YDJ1}$*-GFP-RAS2-CVIM* | this study |
| pWS2049 | *CEN URA3 YDJ1-CIIM* | this study |
| pWS2058 | *CEN HIS3* $P_{CDC43}$*-HsGGT1B* | this study |
| pWS2060 | *CEN URA3 YDJ1-CLVL* | this study |
| pWS2061 | *CEN URA3 YDJ1-CKVL* | this study |
| pWS2062 | *CEN URA3 YDJ1-CLLL* | this study |
| pWS2063 | *CEN URA3 YDJ1-CQLL* | this study |
| pWS2074 | *CEN LEU2 MFA1-SVIA* | this study |
| pWS2075 | *CEN URA3 YDJ1-CLLS* | this study |
| pWS2076 | *CEN URA3 YDJ1-CRII* | this study |
| pWS2077 | *CEN URA3 YDJ1-CRIQ* | this study |
| pWS2078 | *CEN URA3 YDJ1-CRPQ* | this study |
| pWS2079 | *CEN URA3 YDJ1-CSMS* | this study |
| pWS2080 | *CEN URA3 YDJ1-CVLM* | this study |
| pWS2081 | *CEN URA3 YDJ1-CVVS* | this study |
| pWS2087 (MM39) | *CEN URA3 YDJ1-CSQS* | (Berger et al., 2018) |
| pWS2130 | *CEN URA3 YDJ1-CWLC* | this study |
| pWS2131 | *CEN URA3 YDJ1-CYWQ* | this study |
| pWS2132 | *CEN URA3 YDJ1-CYFY* | this study |
| pWS2133 | *CEN URA3 YDJ1-CPLL* | this study |
| pWS2134 | *CEN URA3 YDJ1-CVWW* | this study |
| pWS2135 | *CEN URA3 YDJ1-CWQT* | this study |
| pWS2136 | *CEN URA3 YDJ1-CWIM* | this study |
| pWS2137 | *CEN URA3 YDJ1-CNMG* | this study |
| pWS2138 | *CEN URA3 YDJ1-CETC* | this study |
| pWS2139 | *CEN URA3 YDJ1-CGGN* | this study |
| pWS2140 | *CEN URA3 YDJ1-CWNA* | this study |
| pWS2141 | *CEN URA3 YDJ1-CNPA* | this study |
| pWS2142 | *CEN URA3 YDJ1-CHLR* | this study |
| pWS2143 | *CEN URA3 YDJ1-CCKP* | this study |
| pWS2144 | *CEN URA3 YDJ1-CKRC* | this study |
| pWS2145 | *CEN URA3 YDJ1-CKGE* | this study |
| pWS2146 | *CEN URA3 YDJ1-CQLP* | this study |

| | | |
|---|---|---|
| pWS2147 | *CEN URA3 YDJ1-CPAA* | this study |
| pWS2148 | *CEN URA3 YDJ1-CGRT* | this study |
| pWS2149 | *CEN URA3 YDJ1-CPMT* | this study |
| pWS2150 | *CEN URA3 YDJ1-CYME* | this study |
| pWS2151 | *CEN URA3 YDJ1-CDYQ* | this study |
| pWS2152 | *CEN URA3 YDJ1-CWFC* | this study |
| pWS2153 | *CEN URA3 YDJ1-CDVY* | this study |
| pWS2154 | *CEN URA3 YDJ1-CWHS* | this study |
| pWS2155 | *CEN URA3 YDJ1-CKDD* | this study |
| pWS2156 | *CEN URA3 YDJ1-CSNY* | this study |
| pWS2157 | *CEN URA3 YDJ1-CSTH* | this study |
| pWS2158 | *CEN URA3 YDJ1-CVVR* | this study |
| pWS2159 | *CEN URA3 YDJ1-CPGH* | this study |
| pWS2160 | *CEN URA3 YDJ1-CFTP* | this study |
| pWS2180 | *CEN URA3 YDJ1-CVVM* | this study |
| pWS2256 | *CEN URA3* $P_{YDJ1}$-*His*$_6$-*DNAJA2*-SAHQ | this study |

Disease Models & Mechanisms • Supplementary information

## Table S5. Oligonucleotides used in this study.

| Oligo | Sequence[a] | Gene target |
|---|---|---|
| oWS926 | CCGTTGATGGAACCTTGTGC | RAM2 |
| oWS927 | TGAACGTCGCCATGACAAGAC | RAM2 |
| oWS966 | GATTCCGATGAAGAAGAACAAGGTGGCGAAGGTGTTCAATGTGTA CTCCTCTGATTTTCTTGATAAAAAAAGATCAAC | YDJ1-CVLL |
| oWS986 | GATTCCGATGAAGAAGAACAAGGTGGCGAAGGTGTTCAATGCNN NNNNNNNTGATTTTCTTGATAAAAAAAGA | YDJ1-CRV |
| oWS1001 | TCCGATGAAGAAGAACAAGGTGGCGAAGGTGTTCAATGTAAGCA GCAATGATTTTCTTGATAAAAAAAGATCA | YDJ1-CKQQ |
| oWS1004 | AATTCTACATCTTCCAACAACAATAATAAACGTCCAAAGATGGGA TCCACCATGGCTAACGT | $P_{YDJ1}$-DNAJA2 |
| oWS1005 | AATTCTACATCTTCCAACAACAATAATAAACGTCCAAAGATGCAT CATCACCATCACCATGGA | $P_{YDJ1}$-$His_6$-DNAJA2 |
| oWS1006 | ATGAATCGTGAATAAGTTGATCTTTTTTTATCAAGAAAATTACTGA TGGGCACACTGCACT | $P_{YDJ1}$-$His_6$-DNAJA2 |
| oWS1024 | GATTCCGATGAAGAAGAACAAGGTGGCGAAGGTGTTCAATGTCCC CAACAATGATTTTCTTGATAAAAAAAGATCA | YDJ1-CPQQ |
| oWS1026 | GATTCCGATGAAGAAGAACAAGGTGGCGAAGGTGTTCAATGTGCA ATCATGTGATTTTCTTGATAAAAAAAGATCA | YDJ1-CAIM |
| oWS1027 | GATTCCGATGAAGAAGAACAAGGTGGCGAAGGTGTTCAATGTGCT AAATCTTGATTTTCTTGATAAAAAAAGATCA | YDJ1-CAKS |
| oWS1029 | GATTCCGATGAAGAAGAACAAGGTGGCGAAGGTGTTCAATGTAA GACACAATGATTTTCTTGATAAAAAAAGATCA | YDJ1-CKTQ |
| oWS1030 | GATTCCGATGAAGAAGAACAAGGTGGCGAAGGTGTTCAATGTAA GGTTCAATGATTTTCTTGATAAAAAAAGATCA | YDJ1-CKVQ |
| oWS1031 | GATTCCGATGAAGAAGAACAAGGTGGCGAAGGTGTTCAATGTTTG ATCATGTGATTTTCTTGATAAAAAAAGATCA | YDJ1-CLIM |
| oWS1034 | GATTCCGATGAAGAAGAACAAGGTGGCGAAGGTGTTCAATGTTCT ATCATGTGATTTTCTTGATAAAAAAAGATCA | YDJ1-CSIM |
| oWS1036 | GATTCCGATGAAGAAGAACAAGGTGGCGAAGGTGTTCAATGTGTT TTGTCTTGATTTTCTTGATAAAAAAAGATCA | YDJ1-CVLS |
| oWS1042 | TCCGATGAAGAAGAACAAGGTGGCGAAGGTGTTCAATGTTCTCAA GGTCCATGATTTTCTTGATAAAAAAAGATCA | YDJ1-CSQGP |
| oWS1221 | AACTATATTATCAAAGGTGTCTTCTGGGACCCAGCATGTTTGATTA TGTAGTTTCTGCGTACAAAAACGCGT | MFA1-CLIM |
| oWS1222 | AACTATATTATCAAAGGTGTCTTCTGGGACCCAGCATGTGCTATTA TGTAGTTTCTGCGTACAAAAACGCGT | MFA1-CAIM |
| oWS1256 | GATTCCGATGAAGAAGAACAAGGTGGCGAAGGTGTTCAATGTGTT ATTTTGTGATTTTCTTGATAAAAAAAGATCA | YDJ1-CVIL |
| oWS1257 | CGCGGTGGCGGCCGCTCTAGAACTAGTGGATCCCCCGGGTTTTAC TGCCGAAGGTAAATGTAAA | RAM1 |
| oWS1258 | CTGGGTACCGGGCCCCCCCTCGAGGTCGACGGTATCGATTGTTTA AAGGGTATGTTTAAATCGC | RAM1 |
| oWS1259 | CGCGGTGGCGGCCGCTCTAGAACTAGTGGATCCCCCGGGAGTTTG TCTCCCTCACTGTTTTTA | CDC43 |
| oWS1260 | CTGGGTACCGGGCCCCCCCTCGAGGTCGACGGTATCGATAATACC AGGATGTCGGAATTGATAT | CDC43 |
| oWS1308 | CAGCATATAATCCCTGCTTTA | YDJ1-$CX_{20}X_{20}X_{20}$ |
| oWS1330 | AACTATGATTCCGATGAAGAAGAACAAGGTGGCGAAGGTGTACA ATCTGCATCTCAATGATTTTC | YDJ1-BsrGI-SASQ |

Disease Models & Mechanisms • Supplementary information

| | | |
|---|---|---|
| oWS1359 | TATGATTCCGATGAAGAAGAACAAGGTGGCGAAGGTGTACAATGT/ITRIMIX20//ITRIMIX20//ITRIMIX20/TGATTTTCTTGATAAAAAAAGATC | YDJ1-CX20X20X20 |
| oWS1422 | TCCGATGAAGAAGAACAAGGTGGCGAAGGTGTTCAATGTACTATTTTGTGATTTTCTTGATAAAAAAAGATC | YDJ1-CTIL |
| oWS1424 | TCCGATGAAGAAGAACAAGGTGGCGAAGGTGTTCAATGTAAATGTTATTGATTTTCTTGATAAAAAAAGATC | YDJ1-CKCY |
| oWS1443 | TCCGATGAAGAAGAACAAGGTGGCGAAGGTGTTCAATGTACTATTATTTGATTTTCTTGATAAAAAAAGATC | YDJ1-CTII |
| oWS1460 | TCCGATGAAGAAGAACAAGGTGGCGAAGGTGTTCAATGTCATGCTTGATTTTCTTGATAAAAAAAGATC | YDJ1-CHA |
| oWS1463 | AACCCTCACTAAAGGGAACAAAAGCTGGAGCTCCACCGCGGGAAAAAGGAAGTGTTTCCCTCC | *Hs*FNTA |
| oWS1464 | TCATCATGACGCTATCCATATGCAACTAGAGACCTGCTGGGAGAAAAAGGAAGTGTTTCCCTC | *Hs*FNTA |
| oWS1465 | ACCTTGAGCAGCTTCACCAACACCTTCAGTAGCAGCCATGAATTCTAGATTTGTTGTAAAAAG | *Hs*FNTA |
| oWS1466 | CCAAGCAAGGGTCCATATCATCGCCGTTCTTATGCAGAAAGAAAAAGGAAGTGTTTCCCTCCT | *Hs*FNTB |
| oWS1468 | GATGGTGGACAATAATAAGTAAAAGAAGATGGAGAAGCCATGAATTCTAGATTTGTTGTAAAAAG | *Hs*FNTB |
| oWS1476 | TCCGATGAAGAAGAACAAGGTGGCGAAGGTGTTCAAAGCCATGCTTGATTTTCTTGATAAAAAAAGATC | YDJ1-SHA |
| oWS1500 | GAATTGGAGCTCCACCGCGGTGGCGGCCGCTCTAGAACTAGTGAAAAAGGAAGTGTTTCCCTCC | $P_{PGK1}$-*Hs*PGGT1B |
| oWS1501 | TTCACCAGAACCAGCCAATCTTTCATCTTCAGTAGCAGCCATGAATTCTAGATTTGTTGTAAAAAG | $P_{PGK1}$-*Hs*PGGT1B |
| oWS1604 | TCCGATGAAGAAGAACAAGGTGGCGAAGGTGTTCAATGTATTATTATGTGATTTTCTTGATAAAAAAAGATC | YDJ1-CIIM |
| oWS1606 | GAAGCCTCCAAGAGCGGATCGGGTGGCTGTTGTATTATTATGTGATTTTCTTGATAAAAA | $P_{YDJ1}$-GFP-RAS2-CIIM |
| oWS1607 | TTTTTATCAAGAAAATCACATAATAATACAACAGCCACCCGATCCGCTCTTGGAGGCTTC | $P_{YDJ1}$-GFP-RAS2-CIIM |
| oWS1608 | GAAGCCTCCAAGAGCGGATCGGGTGGCTGTTGTGTTGTTATGTGATTTTCTTGATAAAAA | $P_{YDJ1}$-GFP-RAS2-CVVM |
| oWS1609 | TTTTTATCAAGAAAATCACATAACAACACAACAGCCACCCGATCCGCTCTTGGAGGCTTC | $P_{YDJ1}$-GFP-RAS2-CVVM |
| oWS1610 | GAAGCCTCCAAGAGCGGATCGGGTGGCTGTTGTGTTTTGTCTTGATTTTCTTGATAAAAA | $P_{YDJ1}$-GFP-RAS2-CVLS |
| oWS1611 | TTTTTATCAAGAAAATCAAGACAAAACACAACAGCCACCCGATCCGCTCTTGGAGGCTTC | $P_{YDJ1}$-GFP-RAS2-CVLS |
| oWS1612 | GAAGCCTCCAAGAGCGGATCGGGTGGCTGTTGTGTTATTATGTGATTTTCTTGATAAAAA | $P_{YDJ1}$-GFP-RAS2-CVIM |
| oWS1613 | TTTTTATCAAGAAAATCACATAATAACACAACAGCCACCCGATCCGCTCTTGGAGGCTTC | $P_{YDJ1}$-GFP-RAS2-CVIM |
| oWS1618 | TCCGATGAAGAAGAACAAGGTGGCGAAGGTGTTCAATGTTTGGTTCTATGATTTTCTTGATAAAAAAAGATC | YDJ1-CLVL |
| oWS1619 | TCCGATGAAGAAGAACAAGGTGGCGAAGGTGTTCAATGTAAAGTTCTATGATTTTCTTGATAAAAAAAGATC | YDJ1-CKVL |
| oWS1620 | TCCGATGAAGAAGAACAAGGTGGCGAAGGTGTTCAATGTTTGTTGTTGTGATTTTCTTGATAAAAAAAGATC | YDJ1-CLLL |
| oWS1621 | TCCGATGAAGAAGAACAAGGTGGCGAAGGTGTTCAATGTCAATTGTTGTGATTTTCTTGATAAAAAAAGATC | YDJ1-CQLL |
| oWS1632 | GACAACTATATTATCAAAGGTGTCTTCTGGGACCCAGCTAGCGTTATTGCTTAGTTTCTGCGTACAAAAACG | MFA1-SVIA |

Disease Models & Mechanisms • Supplementary information

| | | |
|---|---|---|
| oWS1633 | TCCGATGAAGAAGAACAAGGTGGCGAAGGTGTTCAATGTTTGTTG TCTTGATTTTCTTGATAAAAAAAGATC | YDJ1-CLLS |
| oWS1634 | TCCGATGAAGAAGAACAAGGTGGCGAAGGTGTTCAATGTAGAATT ATTTGATTTTCTTGATAAAAAAAGATC | YDJ1-CRII |
| oWS1635 | TCCGATGAAGAAGAACAAGGTGGCGAAGGTGTTCAATGTAGAATT CAATGATTTTCTTGATAAAAAAAGATC | YDJ1-CRIQ |
| oWS1636 | TCCGATGAAGAAGAACAAGGTGGCGAAGGTGTTCAATGTAGACC ACAATGATTTTCTTGATAAAAAAAGATC | YDJ1-CRPQ |
| oWS1637 | TCCGATGAAGAAGAACAAGGTGGCGAAGGTGTTCAATGTTCTATG TCTTGATTTTCTTGATAAAAAAAGATC | YDJ1-CSMS |
| oWS1638 | TCCGATGAAGAAGAACAAGGTGGCGAAGGTGTTCAATGTGTTTTG ATGTGATTTTCTTGATAAAAAAAGATC | YDJ1-CVLM |
| oWS1639 | TCCGATGAAGAAGAACAAGGTGGCGAAGGTGTTCAATGTGTTGTT TCTTGATTTTCTTGATAAAAAAAGATC | YDJ1-CVVS |
| oWS1682 | TCCGATGAAGAAGAACAAGGTGGCGAAGGTGTTCAATGTGTTGTT ATGTGATTTTCTTGATAAAAAAAGATC | YDJ1-CVVM |
| oWS1747 | AGCAGCAGCCATCATGGACCTGGAGTGCAGTCCGCTCATCAGTAA TTTTCTTGATAAAAAAAGATC | $P_{YDJ1}$-$HIS_6$-DNAJA2-SAHQ |
| oWS1748 | GATCTTTTTTTATCAAGAAAATTACTGATGAGCGGACTGCACTCCA GGTCCATGATGGCTGCTGCT | $P_{YDJ1}$-$HIS_6$-DNAJA2-SAHQ |

[a]N is any nucleotide; iTrimix20 is a custom mix of 20 trinucleotides utilizing a single codon for each amino acid.

# Supplementary Materials and Methods

## Detailed yeast strain construction strategies

yWS3106 was created by transforming yWS2600 with pWS1651 followed by sporulation and random spore analysis. yWS3109 was created by transforming yWS2601 with pWS1277 followed by sporulation and random spore analysis. yWS3169 was created from yWS3109 transformed with pWS1903 and pWS1934 followed by selection on SC complete solid media containing 1 mg/ml 5-fluoroorotic Acid (5FOA; Research Products International) to eliminate the *URA3* marked plasmid, creating yWS3131, which was subsequently transformed with pWS1623 (BamHI, HindIII, PvuI digest) and selected on YPD-nourseothricin to generate *ydj1::NAT*$^R$. yWS3186 was created in multiple steps. A loop-in loop-out strategy was used to integrate $P_{PGK1}$-*FNTA* at the *RAM1* locus harboring a pre-existing *ram1::KAN*$^R$ disruption. To do this, yWS2542 was transformed with pWS1884 (HpaI digest), selected on SC-uracil to obtain a chromosomal integrant at the *ram1::KAN*$^R$ locus (i.e., loop in), then selected on SC complete solid media containing 5FOA (i.e., loop out) to eliminate the *URA3* marker. Plate-based screening for G418 sensitive candidates yielded yWS3104 that was then transformed with pWS1936 (NsiI digest) and selected on SC-histidine to obtain integrated $P_{PGK1}$-*FNTA* at the *HIS3* locus, generating yWS3134. This strain was backcrossed to BY4742 to obtain the non-petite isogenic strain yWS3186. yWS3202 was created from yWS1632 by two successive

backcrosses to BY4742 to eliminate the petite phenotype (ρ–). yWS3209 was created from yWS2542 by backcross to BY4742 to eliminate the petite phenotype (ρ–). yWS3220 was created in multiple steps in a manner identical to that described for yWS3186, except that the starting strain was yWS1632, and intermediate strains were yWS3105 and yWS3132. yWS3451 was created from yWS3169 by plasmid shuffle.

## Detailed plasmid construction strategies

Various oligonucleotides were used in plasmid constructions (**Table S5**). Plasmids encoding **a**-factor-CaaX variants were created by co-transforming yeast with appropriate PCR-derived DNA fragments and pWS610 (MluI digest) followed by SC-uracil selection. Plasmids encoding Ydj1-CaaX variants were created similarly using pWS1132 (NheI and AflII digest) followed by SC-uracil selection. pWS965 was generated by ligation of a KpnI-SacI fragment encoding *Hs*RCE1 from pWS335 into the same sites of pRS416. pWS1277 was derived by subcloning a PCR-derived fragment encoding the *RAM2* gene, including 439 bp 5´ UTR and 637 bp 3´ UTR, into pRS416. pWS1278 was derived identically to pWS1277 but contained 1333 bp 3´ UTR. pWS1424 was generated by co-transforming yeast with a PCR-derived DNA fragment encoding the *HIS*$_6$*-HsDNAJA2* cDNA sequence and pWS1132 (BsaBI and Nhe1 digest) followed by SC-uracil selection. The *HIS*$_6$*-HsDNAJA2* cDNA sequence was PCR amplified from plasmid #19546 with oligos oWS1005 and oWS1006. pWS1651 was derived by subcloning a PCR-derived fragment amplified from BY4241 encoding the *CDC43* gene, including 415 bp 5´ UTR and 500 bp 3´ UTR, into pRS316. pWS1658 was derived by using the BamHI-XhoI fragment of pWS1655 and recombination-based methods for direct gene replacement of *RAM2* in pWS1278 (NheI digest). pWS1660 was derived by using the BamHI-XhoI fragment of pWS1657 and recombination-based methods for direct gene replacement of *CDC43* in pWS1651 (HindIII digest). pWS1659 was derived by using the BamHI-XhoI fragment of pWS1656 and recombination-based methods for direct gene replacement of *RAM1* in pWS1767 (MscI digest). pWS1767 was derived by subcloning a PCR-derived fragment amplified from BY4241 encoding the *RAM1* ORF, 491 bp 5´ UTR, and 275 bp 3´ UTR into pRS316. QuickChange was used to derive pWS1997-pWS2000 from pWS1961 and pWS2256 from pWS1424. Each of the yeast plasmids encoding human prenyltransferase subunits was also engineered for expression driven by the *PGK1* promoter. Using recombination-based methods, the PCR amplified *PGK1* promoter (590 bp) derived from pWS948 was used to replace the orthologous yeast promoters. pWS1861 was derived using pWS1658 (NotI-digest), pWS1862 using pWS1658 (AfeI-digest), pWS1863 using pWS1719 (PstI-digest), and pWS1914 using pWS1660 (BamHI-digest). pWS1861 and pWS1862 behave identically in all functional tests but differ in that pWS1862 retains 243 bp of *RAM2* outlying genomic sequence upstream of the *PGK1* promoter.

Disease Models & Mechanisms • Supplementary information

## Supplementary references

**Ashok, S., Hildebrandt, E. R., Ruiz, C. S., Hardgrove, D. S., Coreno, D. W., Schmidt, W. K. and Hougland, J. L.** (2020). Protein farnesyltransferase catalyzes unanticipated farnesylation and geranylgeranylation of shortened target sequences. *Biochemistry* **59**, 1149-1162.

**Berger, B. M., Kim, J. H., Hildebrandt, E. R., Davis, I. C., Morgan, M. C., Hougland, J. L. and Schmidt, W. K.** (2018). Protein isoprenylation in yeast targets COOH-terminal sequences not adhering to the CaaX consensus. *Genetics* **210**, 1301-1316.

**Berger, B. M., Yeung, W., Goyal, A., Zhou, Z., Hildebrandt, E. R., Kannan, N. and Schmidt, W. K.** (2022). Functional classification and validation of yeast prenylation motifs using machine learning and genetic reporters. *PLoS One* **17**, e0270128.

**Blanden, M. J., Suazo, K. F., Hildebrandt, E. R., Hardgrove, D. S., Patel, M., Saunders, W. P., Distefano, M. D., Schmidt, W. K. and Hougland, J. L.** (2018). Efficient farnesylation of an extended C-terminal $C(x)_3X$ sequence motif expands the scope of the prenylated proteome. *J Biol Chem* **293**, 2770-2785.

**Brachmann, C. B., Davies, A., Cost, G. J., Caputo, E., Li, J., Hieter, P. and Boeke, J. D.** (1998). Designer deletion strains derived from Saccharomyces cerevisiae S288C: a useful set of strains and plasmids for PCR-mediated gene disruption and other applications. *Yeast* **14**, 115-32.

**Cadinanos, J., Varela, I., Mandel, D., Schmidt, W. K., Díaz-Perales, A., López-Otín, C. and JMP, F.** (2003). AtFACE-2, a prenylated-protein protease from *Arabidopsis thaliana* related to Ras converting enzymes. *J Biol Chem* **278**, 42091-7.

**Chen, P., Sapperstein, S. K., Choi, J. D. and Michaelis, S.** (1997). Biogenesis of the *Saccharomyces cerevisiae* mating pheromone **a**-factor. *J Cell Biol* **136**, 251-69.

**Hildebrandt, E. R., Cheng, M., Zhao, P., Kim, J. H., Wells, L. and Schmidt, W. K.** (2016). A shunt pathway limits the CaaX processing of Hsp40 Ydj1p and regulates Ydj1p-dependent phenotypes. *eLife* **5**, e15899.

**Kim, J. H., Hildebrandt, E. R., Sarkar, A., Yeung, W., Waldon, R. A., Kannan, N. and Schmidt, W. K.** (2023). A comprehensive in vivo screen of yeast farnesyltransferase activity reveals broad reactivity across a majority of CXXX sequences. *G3 (Bethesda)* **13**, jkad094.

**Krishnankutty, R. K., Kukday, S. S., Castleberry, A. J., Breevoort, S. R. and Schmidt, W. K.** (2009). Proteolytic processing of certain CaaX motifs can occur in the absence of the Rce1p and Ste24p CaaX proteases. *Yeast* **26**, 451-463.

**Mokry, D. Z., Manandhar, S. P., Chicola, K. A., Santangelo, G. M. and Schmidt, W. K.** (2009). Heterologous expression studies of *Saccharomyces cerevisiae* reveal two distinct trypanosomatid CaaX protease activities and identify their potential targets. *Eukaryot Cell* **8**, 1891-900.

**Plummer, L. J., Hildebrandt, E. R., Porter, S. B., Rogers, V. A., McCracken, J. and Schmidt, W. K.** (2006). Mutational analysis of the Ras converting enzyme reveals a requirement for glutamate and histidine residues. *J Biol Chem* **281**, 4596-605.

Disease Models & Mechanisms • Supplementary information

**Ravishankar, R., Hildebrandt, E. R., Greenway, G., Asad, N., Gore, S., Dore, T. M. and Schmidt, W. K.** (2023). Specific disruption of Ras2 CAAX proteolysis alters its localization and function. *Microbiol Spectr* **11**, e0269222.

**Schey, G. L., Buttery, P. H., Hildebrandt, E. R., Novak, S. X., Schmidt, W. K., Hougland, J. L. and Distefano, M. D.** (2021). MALDI-MS analysis of peptide libraries expands the scope of substrates for farnesyltransferase. *Int J Mol Sci* **22**, 12042.

**Shoemaker, D. D., Lashkari, D. A., Morris, D., Mittmann, M. and Davis, R. W.** (1996). Quantitative phenotypic analysis of yeast deletion mutants using a highly parallel molecular bar-coding strategy. *Nature Genetics* **14**, 450-6.

**Sikorski, R. S. and Hieter, P.** (1989). A system of shuttle vectors and yeast host strains designed for efficient manipulation of DNA in *Saccharomyces cerevisiae*. *Genetics* **122**, 19-27.

**Stein, V., Kubala, M. H., Steen, J., Grimmond, S. M. and Alexandrov, K.** (2015). Towards the systematic mapping and engineering of the protein prenylation machinery in *Saccharomyces cerevisiae*. *PLoS One* **10**, e0120716.
